# Exploring Negative Feedback Mechanisms in the PTEN-ACE Axis: Application of Electrosorb Hydrogel-Based Gene Delivery for Intervertebral Disc Regeneration

**DOI:** 10.7150/ijbs.111898

**Published:** 2025-05-15

**Authors:** Youfeng Guo, Feng Wang, Yu Zhou, Bijun Wang, Chao Wang, Tao Hu, Desheng Wu

**Affiliations:** 1 Department of Spine Surgery, Shanghai East Hospital, School of Medicine, Tongji University, Shanghai 200092, China.; 2 Department of Medical Genetics, School of Medicine, Tongji University, Shanghai 200092, China.; 3 School of Materials Science and Engineering, Shanghai University, Shanghai 200444, China.

**Keywords:** intervertebral disc degeneration, nucleus pulposus, angiotensin-converting enzyme, PTEN, endoplasmic reticulum autophagy

## Abstract

Intervertebral disc degeneration (IDD), along with associated low back pain, stands as a primary cause of disability. The renin-angiotensin-aldosterone system has been linked to IDD; however, the mechanisms underlying this relationship have not been determined. In this study, the role of angiotensin-converting enzyme (ACE), a key synthetase in the system, in IDD and its regulatory mechanism were evaluated. Our findings revealed that downregulating ACE alleviates IDD. Additionally, phosphatase and tensin homolog (PTEN) regulated ACE through tripartite motif-containing 63 (TRIM63)-mediated K48-linked ubiquitination. PTEN dephosphorylated TRIM63, while polo-like kinase 1 (PLK1) phosphorylated TRIM63 at Ser67 and Ser69, two crucial sites for the interaction between ACE and TRIM63. Importantly, this regulatory axis also influenced endoplasmic reticulum autophagy by modulating O-GlcNAc modification, highlighting its significant role in the regulation of IDD. Furthermore, we developed a chitosan-virus electrosorb hydrogel for IDD repair therapy using lentivirus-mediated gene editing. The hydrogel exhibited excellent swelling, degradation, release rates, and biocompatibility. Specific gene editing by the chitosan-virus electrosorb hydrogel could reduce IDD in rats. These findings support the efficacy of modulating the PTEN-ACE pathway and O-GlcNAc modification and the therapeutic value of chitosan-virus electrosorb hydrogels for IDD.

## Introduction

Low back pain is a leading cause of disability and decreased productivity worldwide, affecting more than 80% of people worldwide 1. The remarkably high prevalence of low back pain imposes substantial dual burdens, significantly compromising patients' quality of life while concurrently generating considerable socio-economic consequences on a global scale. Intervertebral disc degeneration (IDD) is the main cause of low back pain. The intervertebral disc (IVD) is characterized by abnormal mechanical loading, immune disorders, metabolic disorders, and oxidative stress 2, 3. However, the molecular mechanisms underlying IDD have not yet been fully elucidated.

The renin-angiotensin-aldosterone system (RAAS) regulates blood pressure, fluid balance, and salt balance 4. Abnormal RAAS activation contributes to the pathogenesis of an array of chronic and acute diseases, including hypertension 5. Renin, functioning as a protease, cleaves angiotensinogen to create angiotensin I (Ang I). Angiotensin-converting enzyme (ACE) transforms Ang I into Ang II, a crucial regulator of the RAAS. Additionally, it operates via the activation of two G-protein-coupled receptors: Ang II receptor type 1 (AT1R) and Ang II receptor type 2 (AT2R). Although various Ang II-producing enzymes have been identified (such as cathepsin and chymotrypsin), ACE is generally considered to be the most important enzyme in the regulation of Ang II production in the RAAS 6. ACE is a peptidyl dipeptidase that is widely distributed in many types of cells, such as nerve tissue, renal tubular basal cells, and endothelial cells. It is highly active in the testis, epididymis, and lungs 7. ACE influences numerous physiological processes, including renal development, male reproductive function, hematopoiesis, immunological responses, and metabolic functions in chronic illnesses 8. It induces smooth muscle contraction, increases vascular permeability, stimulates inflammation, induces fibrosis, and promotes apoptosis via Ang II and AT1R 9. While emerging evidence has implicated dysregulation of RAAS as a potential risk factor in IDD pathogenesis, the specific role of ACE in this pathological process remains unexplored and warrants systematic investigation.

Phosphatase and tensin homolog (PTEN) are a tumor suppressor gene located on human chromosome 10q23^10^. PTEN is a bispecific protein and lipid phosphatase that primarily targets phosphatidylinositol (3,4,5)-triphosphate (PIP3), hydrolyzing it to phosphatidylinositol (4,5)-diphosphate (PIP2) 11. PI3K signaling is inhibited by PTEN via the inhibition of PIP3-dependent processes, such as membrane recruitment and AKT activation, thereby impairing cell survival, growth, and proliferation 12. The tumor suppressor activity of PTEN is mainly attributed to its lipid phosphatase activity, which antagonizes the PI3K/Akt pathway. Moreover, PTEN shows substantial protein phosphatase activity; specifically, it functions as a protein phosphatase for insulin, insulin-like growth factors, and protein tyrosine kinase PTK6 (BRK), which are crucial pathways in cellular differentiation 13. PTEN can modulate both phosphatase-dependent and -independent signaling pathways. Phosphorylation inactivates PTEN, increasing Akt activity. Hypothermia may exert neuroprotective effects through enhanced phosphorylation of PTEN at Ser380, thereby maintaining Akt activity following ischemia 14. It is interesting to note that peroxynitrite, a neurotoxic derivative of nitric oxide, has been shown to increase Akt phosphorylation, which is associated with increased Cys124 oxidation of PTEN 15. As a general rule, posttranslational modifications (PTMs) of PTEN can alter its quality and quantity and thus play a significant role in disease development 16.

In the endoplasmic reticulum (ER) and Golgi apparatus, protein glycosylation is the most prevalent PTM, with a critical role in protein folding, transport, and degradation 17. Glycosylation is an important prerequisite for protein folding, and abnormal glycosylation leads to misfolded protein accumulation and ER stress 18. There are two types of protein glycosylation: *N*-linked and *O*-linked glycosylation 19. In *O*-linked glycosylation, acetylglucosamine (GlcNAc) is conjugated to the oxygen atom of serine or threonine side chains, with a singular pair of enzymes—*O*-GlcNAc transferase (OGT) and *O*-GlcNAc hydrolase (OGA)—responsible for the addition and removal of the monosaccharide *O*-GlcNAc from the protein substrate 20. Various biological processes are affected by *O*-GlcNAc glycosylation, including epigenetic modification, metabolic homeostasis, stress responses, and immune responses. A change in the intracellular environment, such as inflammation and hypoxia, blocks ER functions, resulting in the accumulation of misfolded or unfolded proteins.

In this study, we found that PTEN regulates the K48-linked ubiquitination of ACE mediated by tripartite motif containing 63 (TRIM63). Moreover, we found that PTEN-induced dephosphorylation of TRIM63 and PLK1-induced phosphorylation of TRIM63 are critical for the interaction between ACE and TRIM63. A negative feedback mechanism is also formed when ACE protein expression decreases and PTEN expression increases, decreasing ACE protein levels. Further analyses showed that this feedback loop also regulates ER autophagy by regulating *O*-GlcNAc modification, thus playing an important role in the regulation of IDD. These findings provide new insights into PTEN-ACE axis construction and regulation.

## Methods and Materials

### Clinical sample collection and ethical review

IVD resection and spinal fusion were performed on nine patients suffering from lumbar spinal stenosis. The details of the patients are shown in Table S1. Sagittal T2-weighted magnetic resonance imaging (MRI) was used to determine the IDD grade of each segment before surgery 21. Clinical and animal studies were reviewed and approved by the Ethics Committee of the Shanghai East Hospital Affiliated to Tongji University. Informed consent was obtained from all participants or their guardians for all publications involving the collection of nucleus pulposus (NP) tissues and any potentially identifiable images or data. Additionally, this clinical study was conducted in accordance with the amended Helsinki Declaration. The animal experiment strictly followed the National Research Council's Guide for the care and use of laboratory animals. Sprague-Dawley (SD) rats (3 months old, n = 200, SPF, female, weighing 250-300 g) were provided by SLAC Laboratory Animals, Inc. The laboratory animals are kept in a controlled environment (with unrestricted access to water and a standard diet).

### Cell culture and treatment conditions

The SD rats were injected with 2% pentobarbital sodium (50 mg/kg) peritoneally and killed immediately following anesthesia. A lumbar IVD annulus fibrous (AF) was incised, the NP tissues were removed using eye forceps, 0.1% type II collagenase was added, and the suspension was digested at 37 °C for 4 h before being filtered through sterile nylon membranes. The supernatant was discarded following centrifugation at 1000 rpm for 5 min. As a final step, the cell microspheres were resuspended in standard medium containing F12-DMEM, 10% fetal bovine serum (Gibco, Waltham, MA, USA), and 1% penicillin/streptomycin (Gibco) at a density of 1 × 10^5^ cells/mL. Cells were cultured in 25 cm^2^ cell culture bottles at 37°C and 5% CO2 in a humidified incubator. Every 3 days, the medium was changed and the cells were observed under a microscope. A 0.25% trypsin-EDTA solution was used to digest and pass the cells after fusion. Subsequent experiments were conducted using NP cells of the second and third generations.

For *in vitro* experiments, NP cells were treated with various drugs at the following concentrations: interleukin-1β (IL-1β) (10 ng/mL, RIL1BI; Thermo Fisher, Waltham, MA, USA), Thiamet-G (TMG) (10 μM, HY-12588; MedChemExpress, Monmouth Junction, NJ, USA), *O*-Glycosyltransferase inhibitor (OSMI) (25 mM, HY-119738; MedChemExpress), rapamycin (Rapa) (100 nM, MedChemExpress), and MG132 (20 μM, S1748; Beyotime, Haimen, China). Transfection experiments involving small interfering RNA, plasmids, and lentiviruses are described in the section on transfection. In the cycloheximide (CHX) chase experiment, NP cells were incubated with CHX (50 μg/mL, 66-81-9; Sigma-Aldrich, St. Louis, MO, USA) for a specified period of time (3, 6, and 9 h) for 24 h following transfection with lentiviral vectors or small interfering RNA. Afterwards, a cell sample was collected for western blot (WB) analyses. Antibodies used in this study are reported in Table S2.

### Human NP specimen collection and immunohistochemistry (IHC)

Within 30 min, human NP tissue was collected and fixed with 4% paraformaldehyde. After 12 h, the specimen was washed three times with phosphate-buffered saline (PBS), dehydrated, and embedded in paraffin. Then, 5-µm-thick continuous slices were cut and stored at 4 °C for further analyses. The antigen epitopes of NP tissue sections were exposed by dewaxing, rehydrating, and soaking overnight at 60 °C in citric acid antigen repair solution buffer (10 mM citric acid, pH 6.0). Next, the samples were treated with 3% hydrogen peroxide for 15 min, endogenous peroxidase was inactivated, and samples were washed three times with PBS. The sections were sealed with goat serum for 30 min at room temperature and then incubated overnight at 4 °C with anti-Collagen II (COL2), anti-Aggrecan (ACAN), and anti-ACE antibodies. The sections were then stained with horseradish peroxide-coupled secondary antibodies (Jackson ImmunoResearch Laboratories, West Grove, PA, US), followed by reverse staining with 3, 3-diaminobenzidine and hematoxylin. After dehydration and xylene cleaning, the sections were sealed with a neutral adhesive and images were taken using an inverted Leica microscope (Leica, Wetzlar, Germany). A semi-quantitative analysis (average fluorescence intensity/integrated optical density per area) was conducted using ImageJ 1.8.0.

### Immunoprecipitation (IP)

A cell lysis buffer (P0013, Beyotime) containing PMSF and phosphatase inhibitors was used for IP analyses (P1081, Beyotime). Pre-clearing of the cell lysate with protein A/G agarose was followed by overnight incubation with the specified primary antibody at 4 °C. A/G agarose beads were added on the following day and incubated for 4 h at 4°C. The mixture was resuspended after three washes. To analyze the supernatant, agarose beads were boiled and centrifuged, and the supernatant was analyzed by WB.

### Lentivirus and siRNA transfection

Regions encoding ACE, TRIM63, and PTEN were labeled with Myc, Flag, and His, respectively, and cloned into the PGMLV-SC5-ZsGreen1-Puro vector by GeneChem (Shanghai, China). From Addgene, we obtained plasmids encoding HA-labeled Ubiquitin (Ub) and its mutants (#18712, #121151, #121152, #22902, #17607, #17606, #17605, and #17604). We generated point mutations in *TRIM63, PTEN, PLK1,* and *ACE* using the Quik Change Mutagenesis Kit (Agilent Technologies, Santa Clara, CA, USA). Lentiviral shRNAs targeting *ACE, PTEN*, and *TRIM63* were obtained from GeneChem. The sequences were as follows: ACE-shRNA#1, 5'-TGGACACCCAGAAGGATATTT-3'; ACE-shRNA#2, 5'-CTACCATCAAGCGGATCATAA-3'; PTEN-shRNA#1, 5'-CGTGCAGATAATGACAAGGAA-3'; PTEN-shRNA#3, 5'-CCACAGCTAGAACTTATCAAA-3'; TRIM63-shRNA#1, 5'-CCGCTCTGATCCTCCAGTACA-3'. The plasmids and shRNAs were transfected using Lipo3000 (Invitrogen, Waltham, MA, USA). Lentiviral transduction in NP cells occurs after 40%-60% alignment, followed by lentivirus infection at 20 MOI. The medium was changed every other day following transfection for 12 h. The transduced NP cells were used for further analyses once they had been fused. OGT siRNA was obtained from GeneChem: OGT-SiRNA, 5'TGAGCAGTATTCCGAGAAA3'. Polo-like kinase 1 (PLK1) siRNA was obtained from Cell Signaling Technology (Danvers, MA, USA; siRNA# 1:6,292) and Sigma-Aldrich (siRNA#2: EHU051011). Lipofectamine2000 (Invitrogen) was used for siRNA transfection according to the manufacturer's instructions, and the transfection efficiency was measured with WB.

### β-Galactosidase staining

Senescence-associated β-galactosidase (SA-β-gal) staining was performed on NP cells using the SA-β-gal Staining Kit (C0602; Beyotime) according to the manufacturer's instructions. After the specified treatment, the NP cells were immobilized with 0.2% glutaraldehyde for 15 min at 37 °C. The newly prepared X-gal dyeing solution (pH 6.0) was added overnight at 37 °C after washing with PBS. The cells were washed with PBS after incubation overnight and were then observed under a microscope.

### Immunofluorescence (IF)

The cells or paraffin-embedded sections were fixed with 4% paraformaldehyde at 4 °C for 15 min, followed by incubation with Triton X-100 and 5% goat serum (P0096, C0265; Beyotime). Cells were then incubated overnight at 4 °C with the following primary antibodies: anti-COL2, anti-p21, anti-a disintegrin and metallo-proteinase with thrombospondin motif 4 (ADAMTS4), and anti-p16. After washing with the primary antibody solution, each sample was incubated for 1 h in the dark with FITC Goat Anti-Rabbit IgG or Cy3 Goat Anti-Rabbit IgG (AS007; ABclonal, Woburn, MA, USA). Cells were incubated in DAPI (C1006; Beyotime) for 5 min. All images were obtained using the same microscope (Leica) and quantitative analyses were performed using ImageJ software.

### Histological analyses

The IVD was collected, fixed with 10% formalin for 48 h, decalcified with 10% EDTA (Sigma) for 6 months, and embedded in paraffin wax. As described previously, the IVD paraffin blocks were cut into 5 mm coronal sections containing endplates, AF, and NP. They were stained with hematoxylin and eosin (HE) and safranin-o/fast green (SO). Sections were evaluated using fluorescent microscopy (FV-1000; Olympus, Tokyo, Japan) and histological scores (ranging from 0 points (normal) to 15 points (severe degeneration) 22.

### Radiological evaluation

IVD radiographs were taken at 0, 4, and 8 weeks (48 kV, 10 mA, 60 cm distance). The digital radiographic images were stored and evaluated using the Image Archive and Communication System (PACS), including measurements of vertebral and IVD height. For each level, the disc height index (DHI) was calculated in accordance with the previous methodology. The DHI was calculated by dividing the height of the IVD by the length of the adjacent vertebra. DHI is expressed as %DHI (post-injection DHI/pre-injection DHI). In addition, we used a 3.0T MRI scanner (Philips-Achieva 3.0T, the Netherlands) to determine the level of IDD.

### WB

Total protein was extracted using a column cell protein extraction kit (PC201plus; Epizyme Biomedical Technology, Shanghai, China). A lysate was added to cell culture dishes or ground NP tissues, and the sample was transferred to a pre-cooled purification column and centrifuged for 1 min at 14000-16000 rpm. The protein concentration was determined using the BCA Protein Assay Kit (Beyotime). The Omni-Easy sample was mixed with buffer (LT101L; Epizyme), boiled for 10 min, separated by SDS, and transferred to a polyvinylidene fluoride membrane. As described previously, the membrane was incubated overnight with the primary antibody. After the secondary antibody was applied for 1 h, ECL fluorescence reagent was added, and ImageJ was used to quantify gray values.

### Quantitative Reverse Transcription Polymerase Chain Reaction (qRT-PCR)

NP cells were harvested using Beyotime, and RNA concentrations were determined by spectrophotometry. Reverse transcription of cDNA was achieved using HiScript IV All-in-One Ultra RT SuperMix (R433-01; Vazyme). The Hieff® qPCR SYBR Green Master Mix Kit (11203ES; Yeasen, Shanghai, China) was used for quantitative PCR, and the CFX384TM system was used for analyses of gene expression levels in comparison with *GAPDH* levels. Table S3 shows the primer sequences.

### Transmission Electron Microscopy (TEM)

Following different interventions, NP cells were collected and fixed in glutaraldehyde (2.5%) overnight, treated with osmium tetroxide (1%), and stained with uranyl acetate (2%) for 1 h. Samples were dehydrated with several acetones, embedded in Araldite, cut into slices, stained with uranyl acetate and lead citrate (1%), and scanned using TEM (HT7700; Hitachi, Tokyo, Japan).

### Tunel staining

Apoptosis of NP cells was determined using the Tunel Assay Kit (Vazyme) following treatment under various conditions. Images of samples were obtained using a fluorescence microscope following nuclear inversion. The images were taken from at least three random fields of view and analyzed using ImageJ software.

### Material preparation and characterization

Chitosan (CS, degree of deacetylation 92%, MW = 52 kDa) (CAS: 9012-76-4) and acetic acid (purity ≥ 99.8%) (CAS: 64-19-7) were provided by Sinopharm Group Co., Ltd. (Shanghai, China). Disodium glycerin phosphate pentahydrate (β-GP, purity: 99%) (CAS: 13408-09-8) was obtained from Shanghai Yuanye Biotechnology Co., Ltd. (Shanghai, China). PBS was obtained from Servicebio (Wuhan, China; G4202). Deionized water was prepared in-house for laboratory use. All chemicals were received and used without further purification. We dissolved 2 g of CS in 100 mL of 0.8% (volume/volume) acetic acid solution and stored it at 4 °C in a constant temperature freezer for 30 min. The β-GP solution (10 percent weight/volume) was cooled to 4 °C. Following this, 5 µL of the virus concentrate was mixed with 5 µL of CS solution at 4 °C. Then, 0.11 µL of GP (9:1 ratio of CS to GP) was added to the mixture under continuous stirring at 4 °C. At 37 °C, the pre-hydrogel liquid gradually gelled, yielding H1. Similarly, H2 was obtained by adjusting the concentration of the β-GP solution to 5% (weight/volume) and leaving the rest unchanged. The amount of CS was changed to 1 g without other changes to make H3.

The mixture was scanned at 3°C using a rheometer (HAAKE MARS60; Thermo Scientific) equipped with a flat plate (plate diameter: 20 mm) in the range of 0.01-1000 Hz to measure the relationship between shear rate and viscosity. Similarly, a mixed solution balanced at 3 °C was placed in a rheometer and scanned at 37 °C at constant frequency to determine the relationship between the energy storage modulus and loss modulus of the solution over time. After the gelled sample was stored at -20 °C overnight, it was dried in a freeze dryer for 2 days. After freeze-drying, the hydrogel was subjected to compression tests using a universal testing machine (INSTRON5944; Instron, Norwood, MA, USA) to evaluate the mechanical properties. The compression test was carried out in the vertical direction at a speed of 3 mm/min. The compressive strength of the stent was measured when the compressive strain was 70%. The microstructure of the scaffold was examined using a cold-field emission scanning electron microscope (SEM; Sigma 300, Zeiss, Germany). To characterize the chemical structure of the CS gel formed at 37 °C, the hydrogel was first lyophilized. An X-ray diffraction analysis (XRD; Smart lab, Rigaku, Japan) was performed, using Cu Kα radiation observed at 2θ from 3° to 70°. Fourier transform infrared spectroscopy (FTIR, NicoletiS20, Thermo Scientific) was performed. CS hydrogels without β-GP were prepared and compared based on FTIR spectra. Swelling rates were measured by immersing freeze-dried scaffolds in PBS (pH 7.4) and maintaining them at 37 °C for 1, 2, 3, and 4 h. The holder was carefully removed after the specified duration and gently wiped with filter paper to remove excess liquid; the new weight (W1) was then determined. The expansion rate (SR%) was calculated using the formula: SR (%) = (W1-W0)/W0 × 100. We assessed degradation rates by soaking freeze-dried scaffolds (initially weighed at W0) in PBS containing lysozyme (1.5 μg/L). To maintain enzyme activity, the scaffold was immersed in a PBS solution containing lysozyme at 37 °C, which was changed daily. Following soaking for 1, 3, 5, 7, 14, or 21 days, the scaffolds were extracted and cleaned three times with deionized water to remove salt and remaining lysozyme. We then freeze-dried the scaffolds and weighed them (W2). The degradation rate is calculated using the formula DR (%) = (W0-W2)/W0 × 100. A virus titer kit (OBiO, Shanghai, China) was used to quantify the amount of virus released from the hydrogel after it was dispersed in PBS in a shaking incubator. A fixed volume of 500 µL was taken at a time to determine the viral concentration released. The precipitation conditions were maintained and fresh PBS was obtained. Briefly, 500 µL of crosslinked composite hydrogel and 500 µL of PBS were mixed together. For 30 days, the PBS was collected and replaced every 5 days. During the preparation of the leaching solution, the composite hydrogel was soaked in the complete medium for 48 h. In accordance with the manufacturer's instructions, the Calcein/PI Cell Viability/Cytotoxicity Assay Kit (C2015S, Beyotime) was used to assess the effect of the leaching liquid on the proliferation of cells in 1, 3, and 6 days. The hemolysis rate test was employed to assess the blood compatibility of biomaterials. The percentage of hemolysis was calculated by referencing complete hemolysis, which was achieved using 1% Triton X-100. The visceral indexes and histopathology were investigated for systemic toxicity evaluation.

### Construction of a rat IDD model

In this study, 200 3-month-old female SD rats were purchased from SLAC Laboratory Animal (Shanghai, China). A ventilated environment with a photoperiod of 12:12 h and a constant temperature of 22 °C was provided for the experimental animals. A model of rat tailbone IDD was established using acupuncture. A 1% pentobarbital sodium solution (0.4 mL/kg, intraperitoneal injection) was used to anesthetize the rats. The tailbone IVD (experimental area: Co6/7) was X-rayed and percutaneously punctured using a 21G needle to a depth of 5 mm, after which the needle was rotated 360°, held for 30 s, and then removed. Subsequently, a 31G sterile needle was inserted to transplant various components. Approximately 2 µL of fluid was injected into each IVD, the microtubules were held for 5 min after injection to prevent fluid leakage. Subcutaneous injections of painkillers were administered to the rats for 3 consecutive days to reduce postoperative pain. We divided the rats into the following groups based upon their treatment: blank control, IDD, hydrogel (hydrogel injection only), hydrogel+shACE, hydrogel+shACE+OSMI (25 μM), hydrogel+shPTEN, hydrogel+shPTEN+OSMI (25 μM), and hydrogel+shACE+shPTEN. After a predetermined period of time (4 and 8 weeks after intervention), a variety of IVD tissues were obtained from the rat tail during rat sacrifice. We conducted histological examinations and IF staining to determine the effect of the above treatments on IVD repair (4 and 8 weeks after intervention).

### Gene Ontology (GO) and Kyoto Encyclopedia of Genes and Genomes (KEGG) pathway enrichment analyses

We used the R software package ClusterProfiler for GO and KEGG pathway enrichment analyses of up- and downregulated differentially expressed genes (DEGs). A threshold of p < 0.05 was used for pathway enrichment and functional filtering for DEGs.

### Statistical analysis

The experiments were repeated at least three times. Statistical analyses were carried out using SPSS 25.0 software (IBM Corp., Armonk, NY, USA). The results were compared using one-way ANOVA and Tukey multiple comparison tests. Values of p < 0.05 were considered statistically significant. A figure of the propose mechanism was created using BioRender software (BioRender, Toronto, Canada).

## Results

### ACE expression was enhanced with degeneration

**Figure 1:**
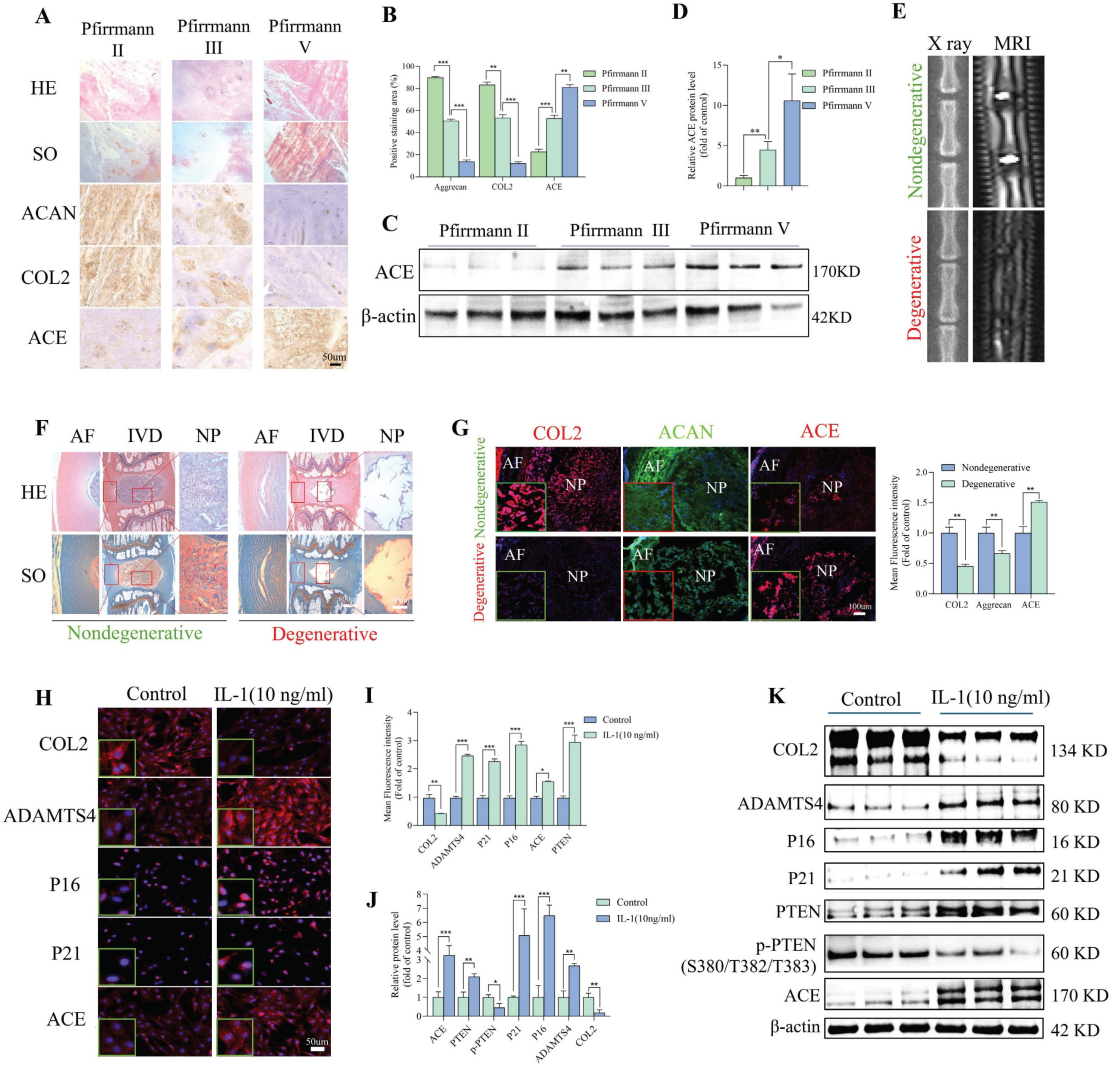
Enhanced expression of ACE in degenerated NP tissues and IL-1-induced NP cells. A-D: Representative images of HE, SO, and immunohistochemical staining for extracellular matrix components (COL2 and ACAN) and ACE demonstrated the histological assessment of human NP tissues exhibiting varying degrees of degeneration. Immunohistochemical staining with ACE antibody and western blot analysis demonstrated the expression levels of ACE protein across each group. Immunohistochemical semi-quantification of ACE (n = 3). The relative band density is measured (n = 3). Scale bar: 50 μm. E-G: Representative images of degenerative and non-degenerative NP tissues from rats, including X-ray, MRI, and histopathology (HE and SO), as well as immunofluorescence staining for ACE and extracellular matrix components (COL2 and ACAN). The average relative optical density was determined (n = 3). Scale bars: 600 μm or 100 μm. H-K: NP cells are subjected to IL-1 (10 ng/ml, 72 h). NP cells were marked with anti-ACE and degeneration markers antibodies (COL2 and ADAMTS4) as well as senescence markers antibodies (P21 and P16), exhibiting representative fluorescence pictures. The western blot analysis demonstrated the expression levels of ACE, PTEN, and phosphorylated PTEN proteins. The relative band density and mean optical density were measured (n = 3). DAPI staining of nuclei; Scale bar: 50 μm. All experiments were repeated three times. The data are presented as the mean ± SD values. One-way ANOVA and Tukey's multiple comparisons test were used for statistical analysis. *p < 0.05, **p < 0.01, ***p < 0.001. NP, nucleus pulposus; HE, hematoxylin and eosin; SO, safranin-o/fast green; COL2, Collagen II; ACAN, aggrecan; ADAMTS4, a disintegrin and metallo-proteinase with thrombospondin motif 4; ACE, angiotensin converting enzyme; PTEN, phosphatase and tensin homolog.

To investigate the clinical relevance of ACE in IDD, we collected three mildly degenerative, three moderately degenerative, and three severely degenerative NP tissues according to the Pfirrmann classification system. Table S1 provides basic patient information. Figure 1A shows representative HE and SO staining images for patients with different grades of IVD degeneration. IHC staining and quantitative analyses showed that ACE expression levels increased with increasing degrees of IDD (Figure 1A and B). The ACE expression patterns detected by IHC staining were opposite those of other important ECM components, such as COL2 and ACAN. As shown in Figure 1C and D, ACE protein expression was significantly higher in Pfirrmann grade III and V IVD than in Pfirrmann grade II IVD (p < 0.001), indicating a positive correlation between ACE expression and the Pfirrmann grade. The effects of ACE were further investigated using a rat model of acupuncture-induced IDD, confirmed through pathological staining and imaging examination (Figure 1E and F). A quantitative IF analysis showed that ACE levels in IDD rats were higher than those in normal rats (p < 0.05) (Figure 1G), consistent with the results for patient samples. These findings suggest that ACE has a significant impact on IDD. The aggregation of cytokines, such as IL-1β and TNF-α, is an important feature of IDD. Therefore, ACE levels were investigated *in vitro* after NP cells were stimulated with IL-1β (10 ng/mL). In response to 72 h of treatment, ACE expression at the RNA and protein levels increased (p < 0.05) (Figure 1H-K).

### Effect of ACE on the function of NP cells

**Figure 2:**
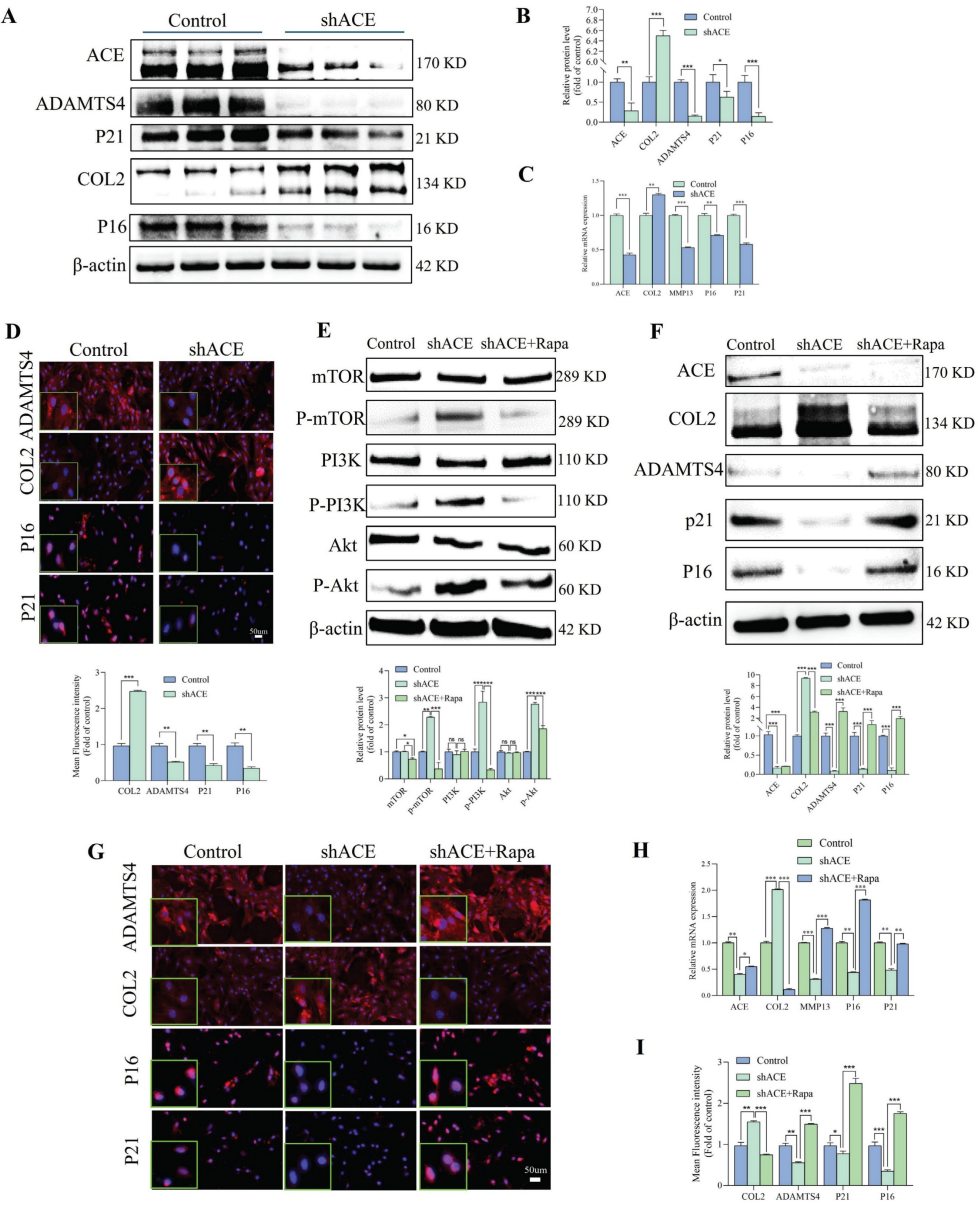
ACE modulates degeneration and senescence in NP cells. A-D: NP cells underwent transduction with the shACE for a duration of 72 hours. Degeneration and senescence-associated proteins COL2, ADAMTS4, P21, and P16 were assessed via western blot analysis, and the relative band density was measured (n = 3). Quantitative PCR was employed to measure RNA levels of degeneration (COL2 and MMP13) and senescence (P21 and P16) markers (n = 3). Subsequently, the NP cells were marked with degeneration indicator antibodies (COL2 and ADAMTS4) and senescence antibodies (P21 and P16), exhibiting typical fluorescence pictures. The relative mean optical density was measured (n = 3). Scale bar: 50 μm. E-F: Forty-eight hours after shRNA transfection, NP cells were treated with the mTOR inhibitor (rapamycin, Rapa, 100 nM) for thirty-six hours. Western blot analysis measured the amounts of total and phosphorylated proteins in the PI3K/Akt/mTOR pathway, along with proteins linked to degeneration (COL2 and ADAMTS4) and senescence (P21 and P16), by measuring the relative band density (n = 3). G and H: Thereafter, the NP cells were marked with degeneration indicator antibodies (COL2 and ADAMTS4) and senescence antibodies (P21 and P16), exhibiting typical fluorescence pictures. The relative mean optical density was measured (n = 3). Scale bar: 50 μm. I: Quantitative PCR was employed to measure the RNA levels of the aforementioned intergroup degeneration and senescence markers (n = 3). DAPI staining of nuclei; All experiments were repeated three times. The data are presented as the mean ± SD values. One-way ANOVA and Tukey's multiple comparisons test were used for statistical analysis. *p < 0.05, **p < 0.01, ***p < 0.001. NP, nucleus pulposus; COL2, Collagen II; ADAMTS4, a disintegrin and metallo-proteinase with thrombospondin motif 4; MMP13, matrix metallopeptidase 13; ACE, angiotensin converting enzyme; PI3K/Akt/mTOR, phosphoinositide 3-kinase/protein kinase B/mammalian target of rapamycin; PCR, polymerase chain reaction.

**Figure 3:**
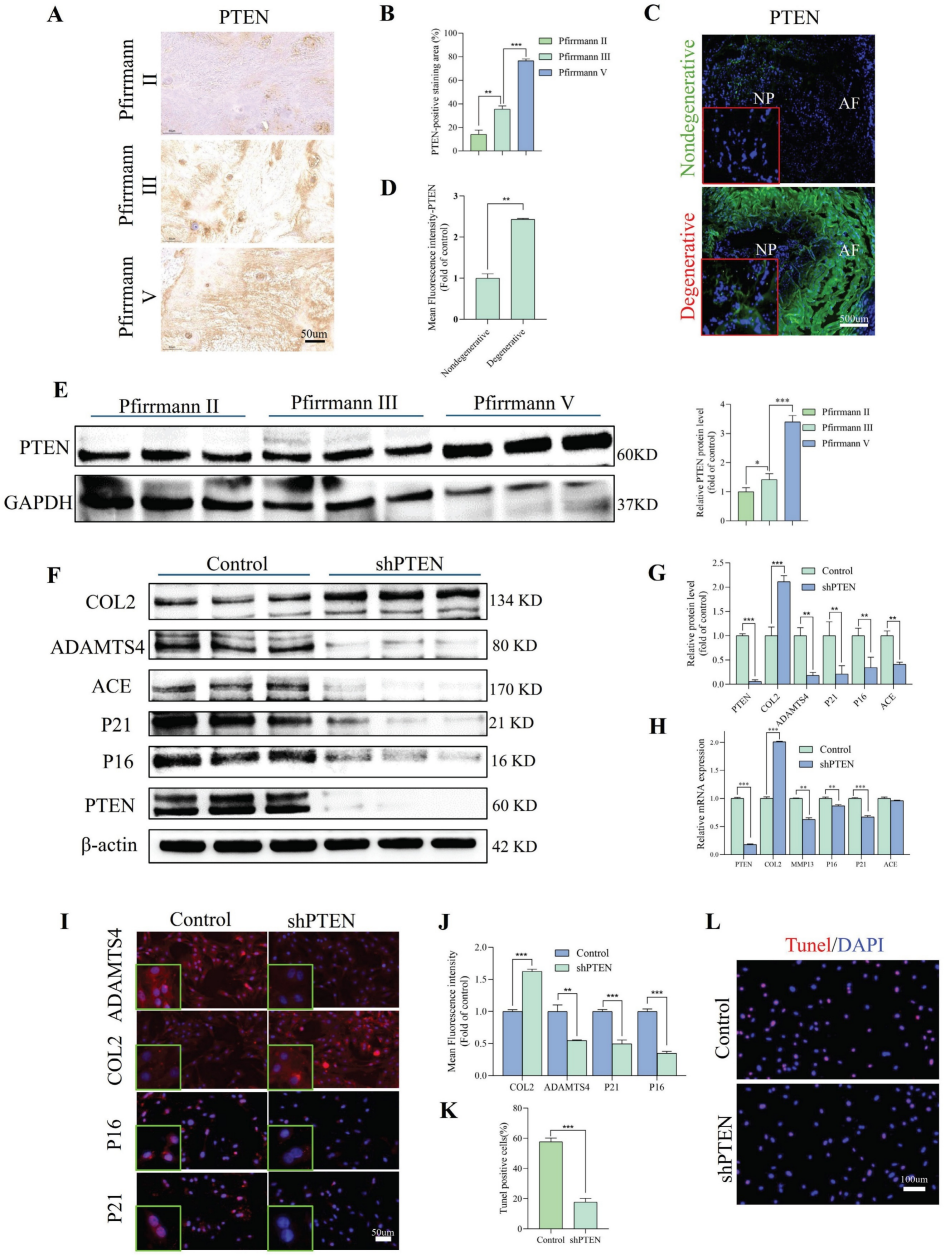
PTEN modulates degeneration and senescence in NP cells. A and B: Immunohistochemical staining revealed the expression levels of PTEN protein in human NP tissues with differing degrees of degeneration (n = 3). Scale bar: 50 μm. C and D: Representative immunofluorescence pictures depicting PTEN in degenerative and non-degenerative NP tissue from rats. The mean relative optical density was ascertained (n = 3). Scale bar: 500 μm. E: Western blot analysis revealed the expression levels of PTEN protein in human NP tissues with differing degrees of degeneration (n = 3). F and G: NP cells were transduced with the shPTEN lentiviral vector for 72 hours. Degeneration and senescence-associated proteins COL2, ADAMTS4, p21, and p16 were evaluated using western blot analysis. Band density was assessed by semiquantitative analysis (n = 3). H: Quantitative PCR was utilized to quantify RNA levels of degeneration (COL2 and MMP13) and senescence markers (P21 and P16) (n = 3). I and J: Degeneration and senescence-associated proteins COL2, ADAMTS4, p21, and p16 were evaluated using immunofluorescence labeling. Fluorescence intensity was assessed by semiquantitative analysis (n = 3). Scale bar: 50 μm. K and L: TUNNEL staining of NP cells was subjected to shRNA transduction. TUNNEL staining positive cells rate was assessed by semiquantitative analysis (n = 3). Scale bar: 100 μm. All experiments were repeated three times. The data are presented as the mean ± SD values. One-way ANOVA and Tukey's multiple comparisons test were used for statistical analysis. *p < 0.05, **p < 0.01, ***p < 0.001. NP, nucleus pulposus; COL2, Collagen II; MMP13, matrix metallopeptidase 13; ADAMTS4, a disintegrin and metalloproteinase with thrombospondin motif 4; PTEN, phosphatase and tensin homolog; PCR, polymerase chain reaction.

We used lentivirus transfection to achieve ACE knockdown in NP cells to better understand the role of this protein in the pathogenesis of IDD. Both q-PCR and WB analyses revealed that ACE expression levels decreased after knockdown (p < 0.05) (Figure 2A-C). As a result of ACE knockdown, both protein and RNA levels of NP cell degeneration markers decreased significantly (p < 0.01). Furthermore, the expression levels of the senescence-related proteins p21 and p16 were lower after ACE knockdown than in the control group. The protective effects of ACE knockdown against NP cell degeneration and senescence were confirmed by IF (Figure 2D), SA-β-gal (Figure S1A), and Tunel (Figure S1B) staining. On the basis of these findings, the down-regulation of ACE inhibits the aging process and cellular degeneration. Furthermore, after shACE was transferred into NP cells, PI3K/Akt/mTOR protein phosphorylation levels increased (Figure 2E). When Rapa was added following ACE knockdown, we observed a decrease in phosphorylation levels and an increase in degeneration and senescence in NP cells (Figure 2E-F). These findings were supported by qPCR, IF, SA-β-gal, and Tunel staining (Figure 2G-I and Figure S1C and D), indicating that ACE can affect NP cell degeneration and senescence by regulating the PI3K/Akt/mTOR pathway. To evaluate upstream regulators of ACE, we focused on PTEN, a specific regulator of PI3K/Akt. PTEN expression levels were elevated in degenerative disc tissues and NP cells (Figure 3A-E). PTEN contributes to IDD because the degeneration and senescence phenotypes of NP cells can be mitigated by PTEN knockdown (Figure 3F-L, Figure S1E). Of note, ACE protein levels decreased after PTEN knockdown, whereas RNA levels remained constant (Figure 3H).

### PTEN and TRIM63 regulate ACE degradation labeled by Ub molecules

**Figure 4:**
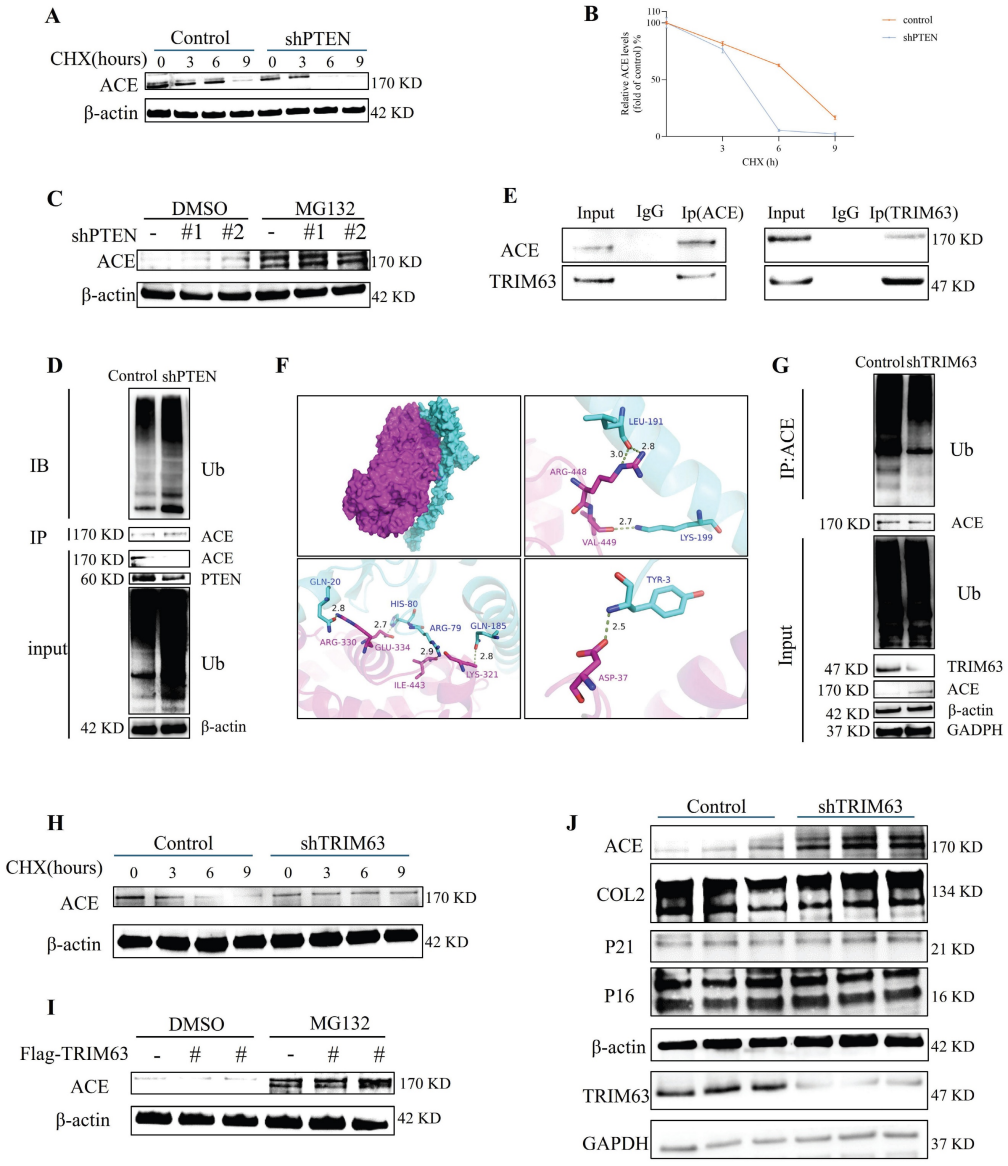
Downregulation of PTEN facilitates ACE degradation and TRIM63 interacts with and stabilizes ACE. A and B: NP cells transfected with shPTEN are treated with cycloheximide (CHX) for a designated duration, after which the cell lysate is subjected to immunoblotting (IB) to determine the relative levels of ACE compared to β-actin. C: MG132 exerts an inhibitory impact on ACE degradation resulting from PTEN knockdown. NP cells were transfection with shRNA for 72 hours and were subsequently treated with MG132 for 6 hours. D: NP cells underwent transfection with shPTEN for a duration of 72 hours. The cell lysate underwent immunoprecipitation (IP) using ACE antibodies, then followed by ubiquitin (Ub) IB. All input lysates were subjected to IB for ACE, PTEN, Ub, and β-actin. E: The interaction between ACE and TRIM63 *in vivo* was confirmed by a co-immunoprecipitation (COIP) experiment. F: Docking of ACE and TRIM63 molecules. The blue protein structure is TRIM63, the purple protein structure is ACE, and the red dotted line is salt bridge. G: NP cells underwent transfection with shTRIM63 for a duration of 48 hours. The cell lysate underwent IP using ACE antibodies, succeeded by Ub IB. All input lysates were subjected to analysis via ACE, TRIM63, Ub, GAPDH, and β-actin IB. H: NP cells transfected with shTRIM63 were treated with CHX for a designated duration, after which the cell lysate was subjected to IB, and the ACE levels were quantified relative to β-actin. I: Flag-TRIM63 was transfected into NP cells for 72 hours, followed by a 6-hour treatment with MG132. J: NP cells transfected with shTRIM63 were analyzed via western blot 72 hours post-transfection. All experiments were repeated three times. NP, nucleus pulposus; ACE, angiotensin converting enzyme; PTEN, phosphatase and tensin homolog; TRIM63, tripartite motif containing 63.

A CHX tracking experiment was conducted to determine the effects of PTEN on ACE stability (Figure 4A, degradation labeled by Ub). The deletion of PTEN accelerated ACE protein degradation significantly. A reduction in ACE protein abundance was observed in NP cells after PTEN knockdown, and this effect was reversed by the addition of 20 μM MG132 after 6 h (Figure 4C). As shown in Figure S1F, Ub levels in NP cells increased significantly following PTEN knockdown. Knocking down PTEN increased endogenous ACE ubiquitination levels in NP cells (Figure 4D). A further search for E3 ubiquitin ligases binding to ACE was conducted. TRIM63, an indicator of muscular atrophy of tripartite motif (TRIM) family proteins-sarcopenia, was a candidate because sarcopenia is associated with the pathogenesis of IDD 23. In this regard, we evaluated the contribution of TRIM63 to the stability of ACE. Through co-immunoprecipitation (CoIP) and molecular docking, we confirmed the endogenous interaction between TRIM63 and ACE (Figure 4E and F). We conducted a CHX tracking experiment to determine whether TRIM63 has an effect on ACE stability. The absence of TRIM63 significantly inhibited the ubiquitination level of ACE (Figure 4G) and thereby inhibited ACE degradation (Figure 4H). NP cells were transfected with Flag-TRIM63 WT and treated with MG132. A WB analysis indicated that TRIM63 regulates ACE protein levels via the proteasome degradation pathway (Figure 4I). TRIM63 knockdown did not significantly alter the degeneration and senescence phenotypes of NP cells (Figure 4J). These data suggest that TRIM63 interacts with ACE and regulates the balance of ACE proteins.

### PTEN regulates ACE ubiquitination through TRIM63

**Figure 5:**
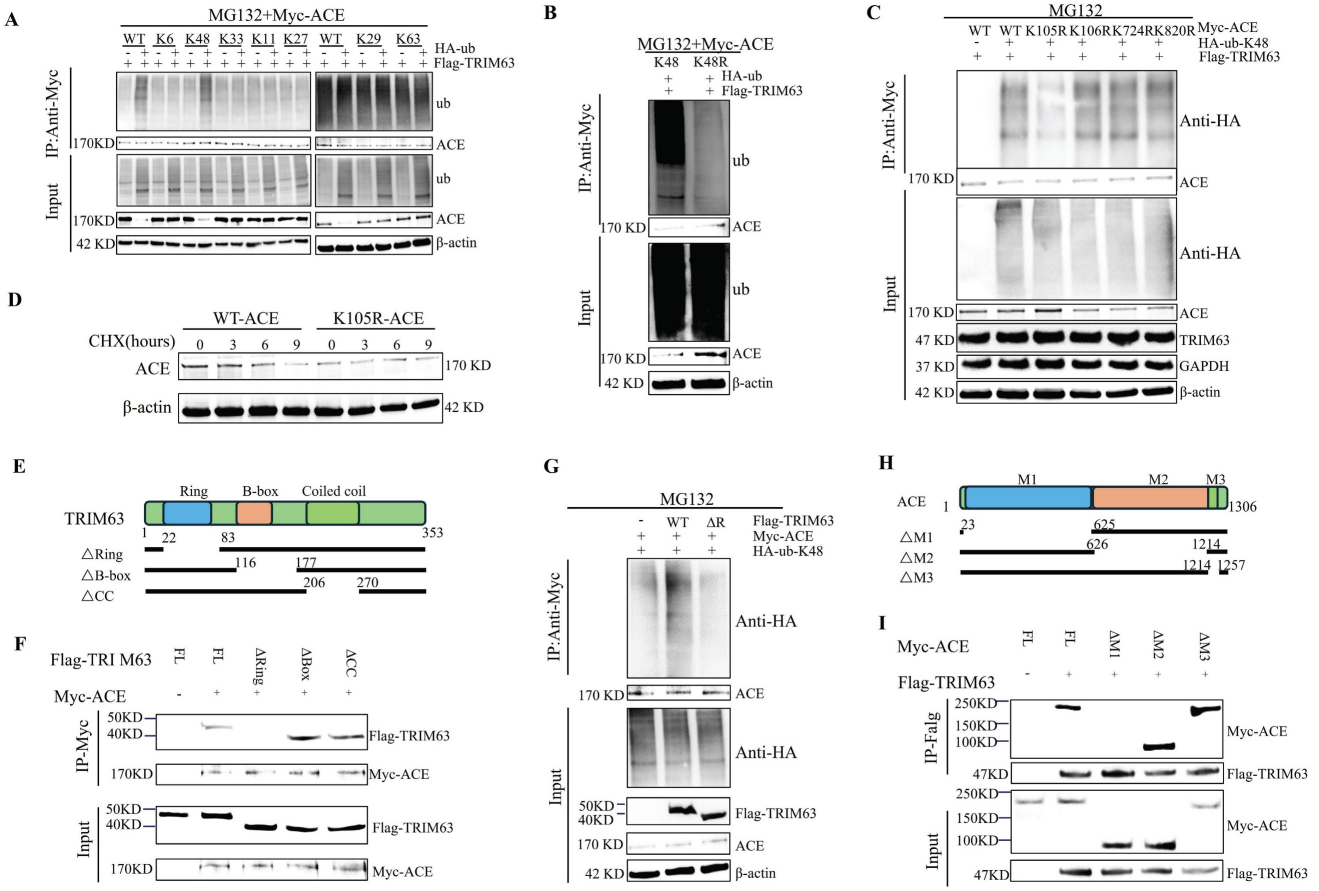
TRIM63 induces the K48-linked ubiquitination transition of ACE. A. NP cells were transfected with Myc-ACE and the indicated ubiquitin (Ub) mutant together with Flag-TRIM63 and then treated with MG132 (20 µM) for 6 h. Cell lysates were subjected to immunoprecipitation (IP) and immunoblotting (IB) with the indicated antibodies. B: NP cells were transfected with Myc-ACE and Flag-TRIM63 together with Ub WT or Ub K48R mutant. After treatment with MG132 (20 μM) for 6 h, the cells were collected and subjected to IP and western blot assays. C: NP cells were transfected with Flag-TRIM63 and HA-Ub-K48 together with Myc-ACE WT or its mutants and then treated with MG132 (20 µM) for 6 h. Cell lysates were subjected to IP and IB with the indicated antibodies. D: Myc-ACE WT or Myc-ACE K105R was transfected into NP cells. After being treated with cycloheximide (CHX) for the indicated times, the cells were subjected to western blot analysis. E: Sketches of full-length (FL) TRIM63 and three TRIM63 deletion mutants. F: Flag-TRIM63 FL or its deletion mutants were co-transfected with the Myc-ACE into NP cells. Cell lysates were subjected to IP using an anti-Myc antibody and then analyzed by IB. G: Myc-ACE was co-transfected with Flag-TRIM63 WT, or Flag-TRIM63 ΔR into NP cells. After treatment with MG132 (20 μM) for 6 h, the cells were collected and subjected to IP and western blot assays. H: Sketches of full-length (FL) ACE and three ACE deletion mutants. I: Myc-ACE FL or its deletion mutants were co-transfected with the Flag-TRIM63 into NP cells. Cell lysates were subjected to IP using an anti-Flag antibody and then analyzed by IB. NP, nucleus pulposus; ACE, angiotensin converting enzyme; TRIM63, tripartite motif containing 63.

Seven lysines on Ub are involved in ubiquitination (K6, K11, K27, K29, K33, K48, and K63). Consequently, we transfected NP cells with Myc-ACE and Flag-TRIM63 along with Ub-WT or a Ub-mutant. TRIM63 enhanced ubiquitination of the K48 junction (Figure 5A). These findings were verified by overexpressing Myc-ACE and Flag-TRIM63 in NP cells as HA-Ub-K48 or HA-Ub-K48R. In comparison with levels in control cells expressing HA-Ub-K48, cells overexpressing HA-Ub-K48R exhibited significantly lower levels of ACE K48-linked ubiquitination (Figure 5B). Furthermore, the ACE protein contains 17 lysine residues, and we identified four putative ubiquitination sites previously reported on PhosphosSitePlus. A set of mutant ACEs was constructed to investigate whether TRIM63 catalyzes ACE ubiquitination at these sites. To further determine the ubiquitination site of ACE, we mutated each Lys into Arg and co-transfected each ACE mutant into NP cells with HA-Ub-K48 and Flag-TRIM63. Only one mutation (K105R) resulted in a TRIM63-mediated reduction in ubiquitination (Figure 5C). NP cells co-transfected with Flag-TRIM63 and Myc-ACE S105A were used for further analyses. A mutation at the 105th lysine site significantly inhibited ACE degradation (Figure 5D).

To determine the structural requirements for the TRIM63-ACE interaction, we generated three deletion mutants of TRIM63 (ΔR, ΔB, and ΔC) (Figure 5E). Myc-ACE, Flag-TRIM63WT, and Flag-TRIM63 (ΔR, ΔB, and ΔC) were co-transfected into NP cells for anti-Myc antibody IP and WB analyses. It was found that TRIM63ΔR binds to ACE, while TRIM63ΔB and TRIM63ΔC do not, suggesting that the Ring domain of TRIM63 is required for its binding to ACE (Figure 5F). Consistent with these findings, Myc-ACE was heavily ubiquitinated when it was co-expressed with TRIM63WT; however, the degree of ubiquitination decreased when it was co-expressed with the TRIM63 mutant (TRIM63ΔR, lacking ring domains) (Figure 5G). We produced a set of mutants that are deficient in ACE (ΔM1, ΔM2, and ΔM3) (Figure 5H). NP cells were transfected with Myc-ACE (ΔM1, ΔM2, and ΔM3), Myc-ACE, and Flag-TRIM63 for anti-Flag antibody IP and WB analyses. We found that Myc-ACE M1 could bind TRIM63, while Myc-ACE ΔM2 and Myc-ACE ΔM3 could not, suggesting that the M1 domain of ACE is required for binding (Figure 5I). These results demonstrate that TRIM63 mediates the ubiquitination of ACE at K105 by K48, clarifying the structural requirements for the interaction between ACE and TRIM63.

**Figure 6:**
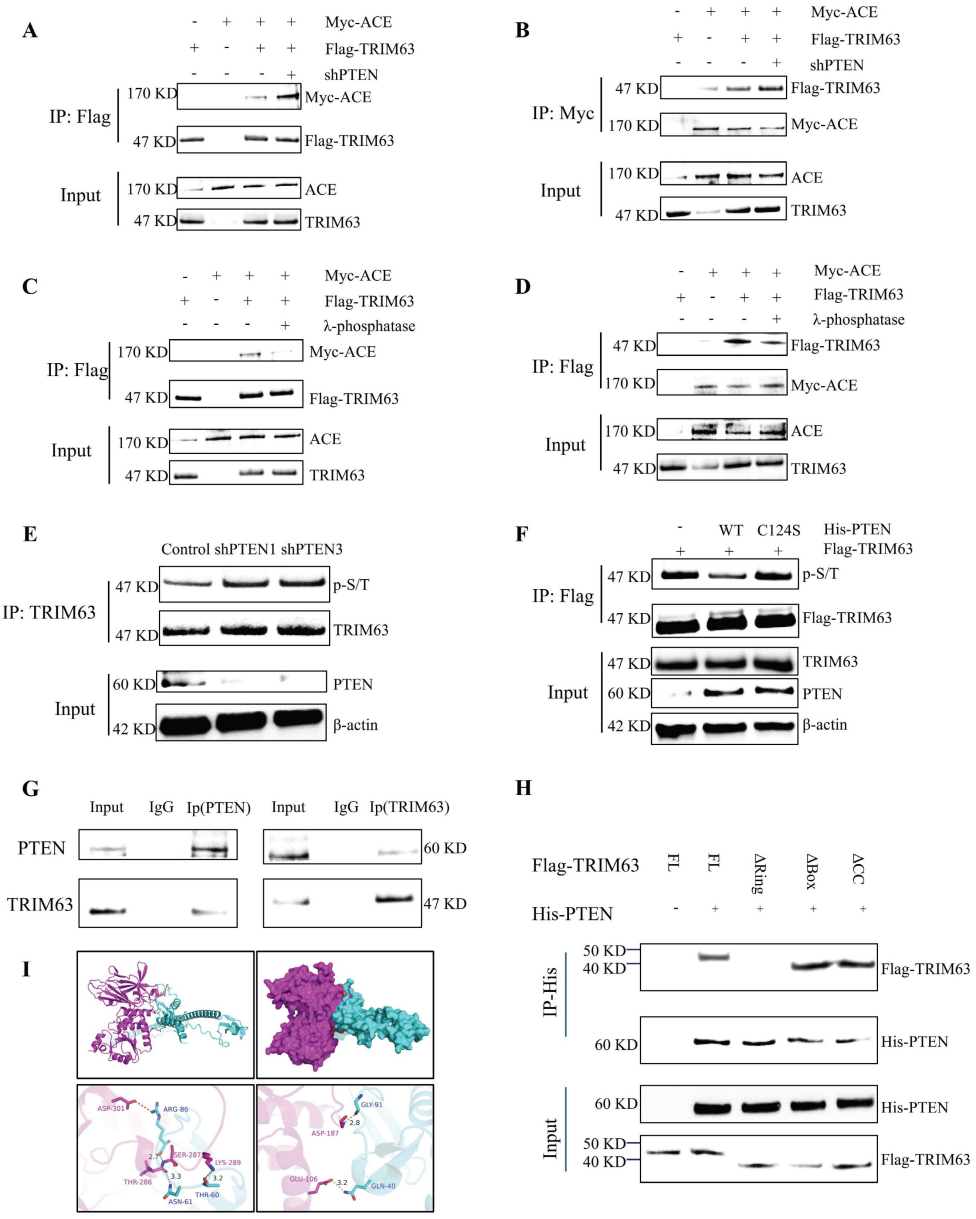
PTEN regulates the ubiquitination of ACE through TRIM63. A-D: IB analysis of cell lysates and anti-Flag (A and C) or anti-Myc (B and D) immunoprecipitates derived from NP cells treated as indicated. All input lysates were subjected to immunoblotting (IB) for ACE and TRIM63. E: NP cells treated with shPTEN were subjected to immunoprecipitation (IP) and IB with the indicated antibodies. All input lysates were subjected to IB for PTEN and β-actin. F: His-PTEN or His-PTEN C124S were co-transfected with the Flag-TRIM63 into NP cells. Cell lysates were subjected to IP using a TRIM63 antibody and then analyzed by IB. G: The interaction between PTEN and TRIM63 *in vivo* was confirmed by immunoprecipitation (COIP). H: Docking of PTEN and TRIM63 molecules. Purple protein structure represents PTEN, sky blue protein structure represents TRIM63, and red dotted lines represent salt bridges. I: Flag-TRIM63 FL or its deletion mutants were co-transfected with the His-PTEN into NP cells. Cell lysates were subjected to IP using an anti-His antibody and then analyzed by IB. NP, nucleus pulposus; ACE, angiotensin converting enzyme; PTEN, phosphatase and tensin homolog; TRIM63, tripartite motif containing 63.

PTEN and ACE do not interact directly, and therefore we analyzed the influence of PTEN on the interaction between ACE and TRIM63 to further understand the mechanism by which PTEN regulates ACE stability. After NP cells were co-transfected with Flag-TRIM63 and Myc-ACE, ACE or TRIM63 antibody IP and WB analyses were conducted. As shown in Figure 6A-B, PTEN knockdown promoted the interaction between ACE and TRIM63. It is possible that PTEN mediates the interaction between ACE and TRIM63 by altering TRIM63 phosphorylation levels. To study the effect of TRIM63 phosphorylation on its binding with ACE, NP cells were co-transfected with Flag-TRIM63 and Myc-ACE, and λ-phosphatase was added to the lysate. ACE or TRIM63 antibody IP and WB analyses showed that a decrease in the phosphorylation level resulted in a weakening of the interaction between ACE and TRIM63 (Figure 6C-D). A lentiviral shPTEN vector was transferred into NP cells for WB and IP analyses. After PTEN knockdown, TRIM63 phosphorylation levels increased (Figure 6E). After NP cells were transfected with phosphatase defect-inactivated His-PTEN C124S and Flag-TRIM63, TRIM63 phosphorylation increased (Figure 6F). We confirmed that TRIM63 and PTEN interact endogenously through CoIP and molecular docking (Figure 6G-H). To determine the structural requirements for the TRIM63-PTEN interaction, NP cells were co-transfected with Myc-ACE, Flag-TRIM63 WT, and Flag-TRIM63 (ΔR, ΔB, and ΔC) for anti-His antibody IP and WB analyses. We found that TRIM63ΔR binds to PTEN, while TRIM63ΔB and TRIM63ΔC do not, suggesting that the Ring domain of TRIM63 is required for its binding to PTEN (Figure 6I).

### Phosphorylation mechanism of TRIM63

**Figure 7:**
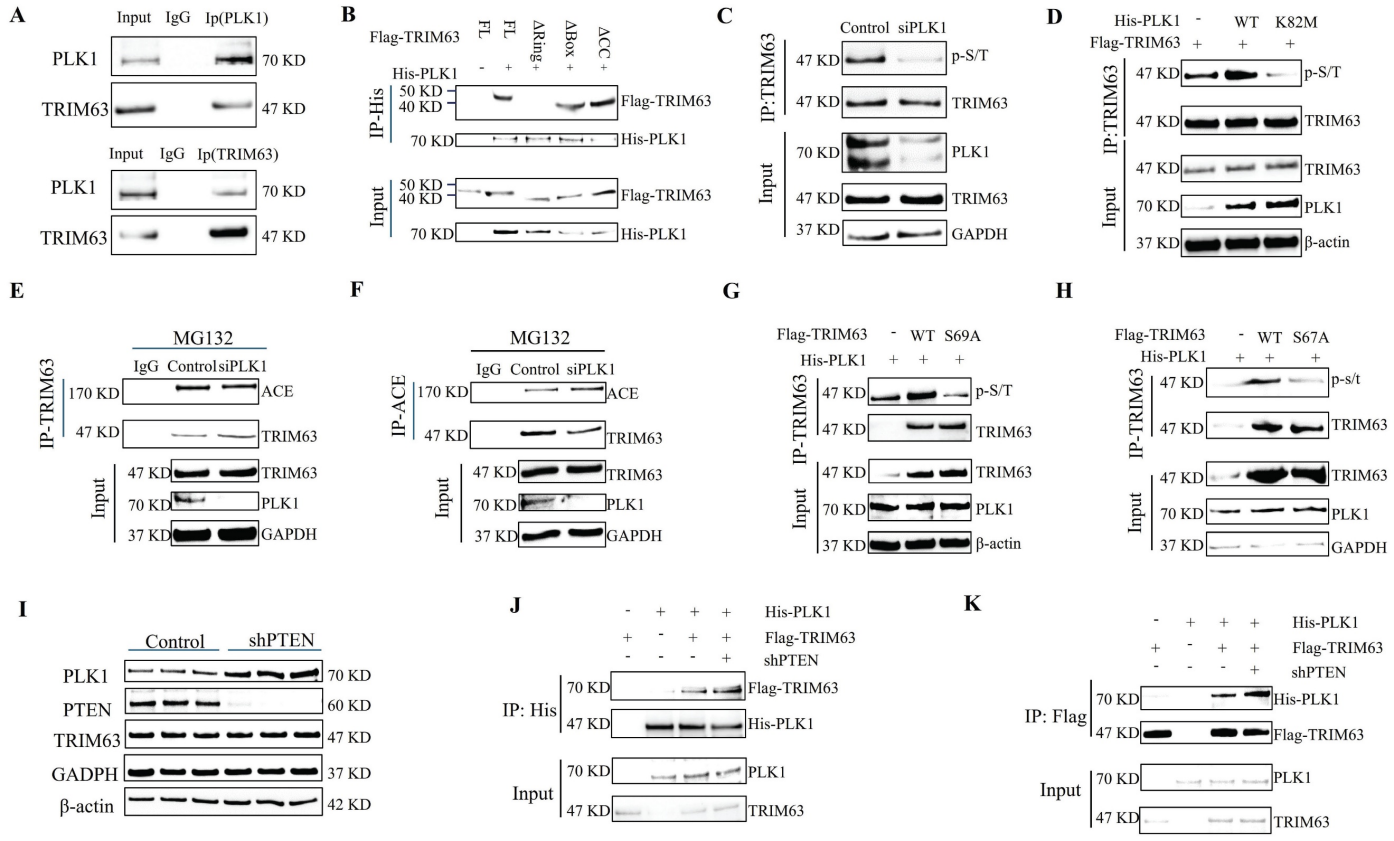
Phosphorylation mechanism of TRIM63. A: The interaction between PLK1 and TRIM63 *in vivo* was confirmed by immunoprecipitation (COIP). B: Flag-TRIM63 full-length (FL) or its deletion mutants were co-transfected with the His-PLK1 into NP cells. Cell lysates were subjected to immunoprecipitation (IP) using an anti-His antibody and then analyzed by immunoblotting (IB). C: NP cells treated with PLK1 siRNAs or not were subjected to IP and IB with the indicated antibodies. D: NP cells transfected with Flag-TRIM63 and His-PLK1 WT or His-PLK1 K82M were subjected to IP and IB with the indicated antibodies. E and F: NP cells transfected with control siRNA or PLK1 siRNA. IB analysis of cell lysates and anti-TRIM63 (E) or anti-ACE (F) immunoprecipitates derived from NP cells treated as indicated. G and H: Flag-TRIM63 WT, Flag-TRIM63 S67A or Flag-TRIM63 S69A was co-transfected with His-PLK1 into NP cells. After that, NP cells treated with MG132 (20 µM) for 6 h. Cell lysates were subjected to IP and IB with the indicated antibodies. I: NP cells were transduced with the shPTEN for 72 hours. Proteins PLK1, and TRIM63, and PTEN were evaluated using western blot analysis. J and K: NP cells treated with shPTEN were subjected to IP and IB with the indicated antibodies (His or Flag). All input lysates were subjected to IB for PLK1 and TRIM63. NP, nucleus pulposus; ACE, angiotensin converting enzyme; PTEN, phosphatase and tensin homolog; TRIM63, tripartite motif containing 63; PLK1, polo-like kinase 1.

For numerous TRIM family proteins, phosphorylation is required for substrate recognition, indicating that their phosphorylated state plays an important role in enzymatic activity 24. To test whether phosphorylation is required for TRIM63 activity, we performed dephosphorylated immunoprecipitation of TRIM63 and ACE through treatment with λ-phosphatase and found that the interaction between TRIM63 and ACE was severely reduced (Figure 6C-D), prompting us to investigate the putative kinase mediating the TRIM63-ACE interaction. We confirmed the endogenous interaction between TRIM63 and PLK1 via CoIP (Figure 7A). To determine the structural requirements for the TRIM63-PLK1 interaction, His-PLK1, Flag-TRIM63 WT, and Flag-TRIM63 (ΔR, ΔB, and ΔC) were co-transfected into NP cells for IP and WB analyses. We found that TRIM63 ΔR was able to bind PLK1, while TRIM63ΔB and TRIM63ΔC were not, suggesting that the Ring domain of TRIM63 is required for its binding to PLK1 (Figure 7B). Using phospho-(Ser/Thr) antibodies for monitoring, a significant reduction in TRIM63 phosphorylation was observed following the siRNA-mediated knockdown of PLK1 (Figure 7C). Overexpression of PLK1 mutants (K82M) led to a reduction in TRIM63 phosphorylation levels (Figure 7D). As a consequence, the interaction between endogenous ACE and TRIM63 was weakened after PLK1 knockdown (Figure 7E-F). Furthermore, we identified two putative TRIM63 phosphorylation sites (Ser67 and Ser69) previously reported on PhosphosSitePlus and that are relatively conserved across species. We then engineered TRIM63 deletion mutants and performed *in vitro* kinase assays to identify phosphorylation sites. TRIM63 phosphorylation levels were decreased at both of the phosphorylation sites above when Serine/threonine (S/T) was replaced with alanine (A). The results of these experiments strongly suggest that PLK1 specifically phosphorylates Ser67 and Ser69 of TRIM63 (Figure 7G-H). In this manner, the phosphorylation levels of TRIM63 are correlated with PLK1 activity, indicating that PLK1 is a key kinase targeting TRIM63. These observations provide evidence that PLK1-mediated phosphorylation of TRIM63 contributes to the interaction between TRIM63 and ACE and thereby to the stability of ACE. Moreover, PLK1 protein expressed increased significantly after the transfection of NP cells with shPTEN (Figure 7I and Figure S2A), and the interaction of PLK1 with TRIM63 was enhanced (Figure 7J and K).

### Negative feedback axis of PTEN-ACE

**Figure 8:**
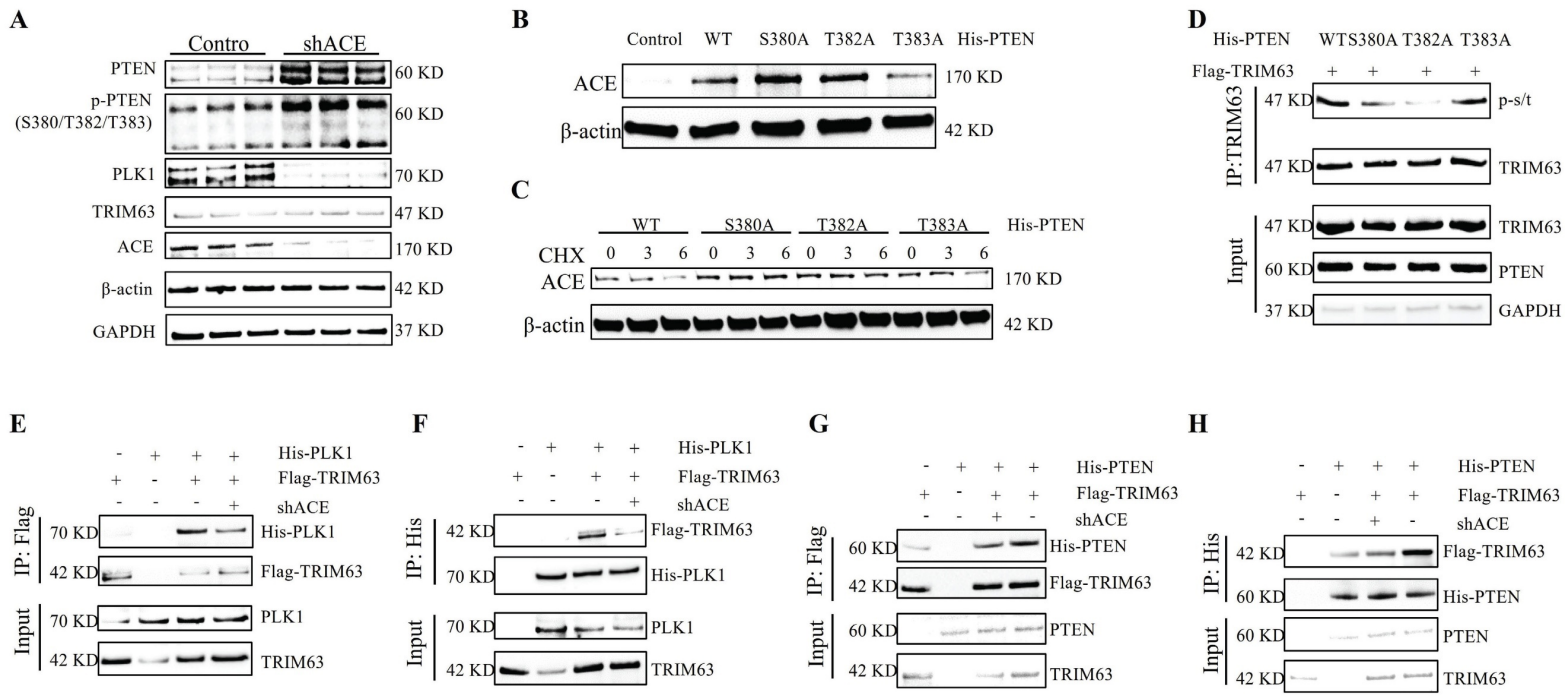
Negative feedback axis of PTEN-ACE. A: NP cells were transduced with the shACE for 72 hours. Proteins PLK1, and TRIM63, phosphorylated PTEN, and PTEN were evaluated using western blot analysis. B: His-PTEN WT, His-PTEN S380A, His-PTEN T382A or His-PTEN T383A was transfected into NP cells and ACE protein expression was analyzed via western blot 72 hours post-transfection. C: NP cells transfected with His-PTEN WT, His-PTEN S380A, His-PTEN T382A or His-PTEN T383A are treated with cycloheximide (CHX) for a designated duration, after which the cell lysate is subjected to immunoblotting (IB) to determine the relative levels of ACE compared to β-actin. D: His-PTEN WT, His-PTEN S380A, His-PTEN T382A or His-PTEN T383A was co-transfected with Flag-TRIM63 into NP cells. Cell lysates were subjected to immunoprecipitation (IP) and IB with the indicated antibodies. E and F: NP cells treated with shACE were subjected to IP and IB with the indicated antibodies (Flag or His). All input lysates were subjected to IB for PLK1 and TRIM63. G and H: NP cells treated with shACE were subjected to IP and IB with the indicated antibodies (Flag or His). All input lysates were subjected to IB for PTEN and TRIM63. NP, nucleus pulposus; ACE, angiotensin converting enzyme; PTEN, phosphatase and tensin homolog; TRIM63, tripartite motif containing 63.

After lentivirus-mediated ACE knockdown in NP cells, levels of PTEN and phosphorylated PTEN increased, with phosphorylation sites located at S380, T382, and T383 (Figure 8A and Figure S2B). The ACE protein level increased after the transfection of NP cells with the phosphorylation site mutant plasmids (His-PTEN S380A and His-PTEN T382A); however, the opposite results were obtained for His-PTEN T383A (Figure 8B). Further CHX tracking experiments showed that the protein decay rate of ACE was slowed down only for His-PTEN S380A and His-PTEN T382A, but not for His-PTEN T383A (Figure 8C). We examined TRIM63 phosphorylation levels following mutant plasmid transfection (His-PTEN S380A, T382A, and T383A), demonstrating that His-PTEN S380A and T382A decreased phosphorylation levels, whereas phosphorylation levels for His-PTEN T383A were comparable to those for the WT (Figure 8D). PLK1 protein levels decreased in NP cells after the knockdown of ACE (Figure 6A). Following ACE knockdown, the strength of the interaction between PLK1 and TRIM63 increased significantly (Figure 8E-F). In this case, PTEN binding to TRIM63 was weakened, resulting in increased phosphorylation of this protein (Figure 8G-H). The magnitude of such negative feedback is constrained and cannot entirely counteract the impact of ACE knock-down. Subsequent investigations revealed that the combined transfection of shACE and shPTEN resulted in markedly decreased levels of degeneration and senescence markers relative to those in the individual shACE or shPTEN transfection groups (Figure S3).

### Effect of the PTEN-ACE axis on O-GlcNAc on ER autophagy

**Figure 9:**
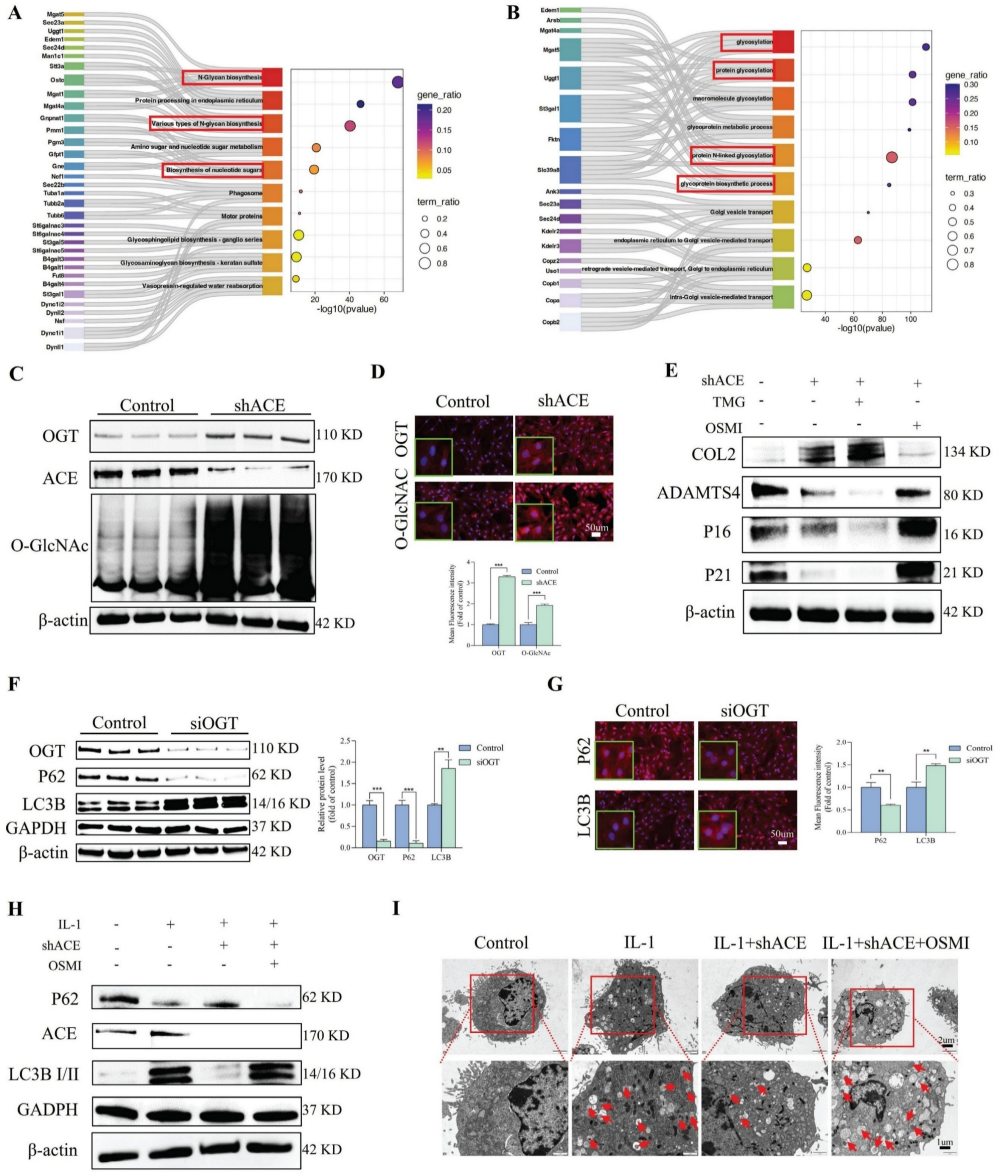
The effect of glycosylation regulated by ACE on ER autophagy. A and B: The Sankey and Bubble plot of metabolic pathway KEGG and GO analysis. C-D: NP cells underwent transduction with the shACE for a duration of 72 hours. OGT and O-GlcNAc were assessed via western blot analysis. Subsequently, the NP cells were marked with OGT and O-GlcNAc antibodies, exhibiting typical fluorescence pictures. The relative mean optical density was measured (n = 3). Scale bar: 50 μm. E: Forty-eight hours after shACE transfection, NP cells were treated with the TMG (10 μM) or OSMI (25 μM) thirty-six hours. Western blot analysis measured the proteins linked to degeneration (COL2 and ADAMTS4) and senescence (P21 and P16) (n = 3). F and G: NP cells transfected with OGT siRNA. P62 and LC3B were assessed via western blot analysis, and the relative band density was measured (n = 3). Subsequently, the NP cells were marked with P62 and LC3B antibodies, exhibiting typical fluorescence pictures. The relative mean optical density was measured (n = 3). Scale bar: 50 μm. H: The NP cells treated with IL-1 (10 ng/ml) were transfected with shACE. The NP cells transfected with shACE were treated with OSMI (25 μM) or not. The levels of P62 and LC3B were detected by western blot (n = 3). I: Autophagosomes/autolysosomes containing ER fragments or whorls were evaluated by transmission electron microscopy. Red arrows represent autophagosomes. Scale bars: 2 μm and 1 μm. All experiments were repeated three times. The data are presented as the mean ± SD values. One-way ANOVA and Tukey's multiple comparisons test were used for statistical analysis. *p < 0.05, **p < 0.01, ***p < 0.001. NP, nucleus pulposus; ACE, angiotensin converting enzyme; ER, endoplasmic reticulum; KEGG, Kyoto encyclopedia of genes and genomes; GO, gene ontology; OGT, O-linked N-acetylglucosamine transferase; O-GlcNAc, O-linked β-N-acetylglucosamine; TMG, Thiamet G; OSMI, O-GlcNAc transferase inhibitor; COL2, Collagen II; ADAMTS4, a disintegrin and metallo-proteinase with thrombospondin motif 4.

Following shACE transfection for 48 h, RNA was extracted from NP cells for a transcriptomic analysis. To explore the biological characteristics of DEGs, we conducted GO and KEGG enrichment analyses; the results are shown in Figure 9A-B and Figure S4. DEGs were involved in several energy-related pathways, including producing precursor metabolites and oxidizing organic compounds for energy. Analyses of glucose metabolism, particularly glycosylation, are critical to understand the role of ACE in the regulation of IDD. ACE knockout increased OGT and O-GlcNAc protein levels in NP cells, as evidenced by IF staining results (Figure 9C and D). Similar increases were not observed after PTEN knockdown (Figure S1G-H). In addition, after adding TMG or OSMI 48 h after ACE knockdown, we observed that the degeneration and senescence indices of NP cells decreased or increased, respectively (Figure 9E and Figure S5A). Furthermore, IF and SA-β-gal staining confirmed that the intensity of each index followed a similar pattern (Figure S6E-H). To further study the regulatory effects of OGT on the activity and function of NP cells, we transferred small interfering siOGT into NP cells for 72 h, resulting in an increase in NP cell degeneration and senescence, as determined through WB and IF staining (Figure S6A-D).

We determined the levels of LC3B and p62, which are associated with ER autophagy. As shown in Figure 9F, LC3B protein expression was significantly higher in the siOGT transfection group than in the control group. This up-regulation was accompanied by a decrease in p62, indicating that ER autophagy was increased by knocking down OGT. Additionally, IF analysis confirmed that siOGT transfection increased LC3B fluorescence intensity while decreasing p62 fluorescence intensity compared with levels in the control group (Figure 9G). NP cells treated with IL-1 (10 ng/mL) exhibited an increase in LC3B protein expression and a decrease in p62 protein expression, and these changes were attenuated by transfection with shACE (Figure 9H and Figure S5B). However, further treatment with OSMI resulted in significantly higher LC3B expression levels and lower p62 expression levels than those for shACE alone. In addition, we examined the formation of autophagosomes containing ER structures by TEM to verify the presence of ER autophagy. As shown in Figure 9I, when treated with IL-1 (10 ng/mL), the number of autophagosomes containing ER fragments increased and the number of autophagosomes increased slightly. There was no significant improvement in the number of autophagosomes or the number of ER-dilated vesicles following shACE treatment. After further treatment with OSMI based on shACE transfection, the number of autophagosomes and ER-dilated vesicles increased significantly. These data suggest that ACE can further regulate ER autophagy by altering glycosylation levels.

### Characterization of hydrogels

**Figure 10:**
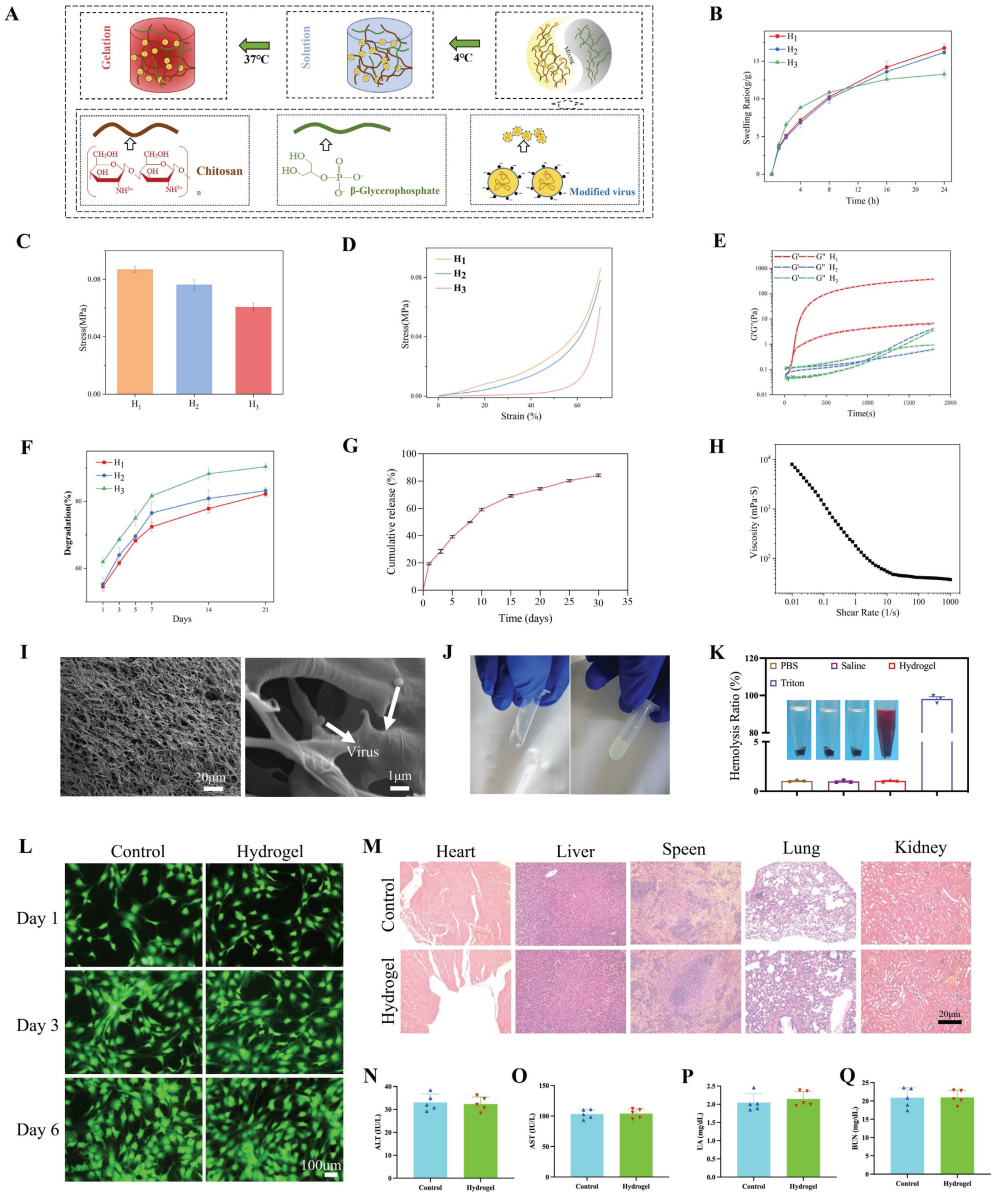
Preparation and characterization of hydrogel. A: Schematic elucidating the preparation of hydrogel. B: Swelling ratios of the prepared hydrogels (H1, H2, and H3). C: The compressive strength of the prepared hydrogels (H1, H2, and H3) at 70% compression. D: Stress-strain curves of the prepared hydrogels (H1, H2, and H3). E: Gelation time of the prepared hydrogels (H1, H2, and H3). F: Degradation ratios of the prepared hydrogels (H1, H2, and H3). G: Dynamic virus release behaviors of the hydrogel (H1). H: Rheological shear strength of the hydrogel (H1). I: Scanning electron microscope images of composite hydrogel loaded with the virus. Scale bars: 100 μm and 1 μm. J: Schematic phase transition of representative hydrogels. K: Cytotoxicity (hemolytic) activity of the hydrogel (H1) in rat red blood cells. L: Cytocompatibility of hydrogels indicated by Live/dead staining on days 1, 3 and 6. Scale bar: 100 μm. M: *In vivo* experimental visceral toxicity analysis. HE staining sections of major organs (heart, liver, spleen, lung and kidney) in each group. Scale bar: 20 μm. N-Q: Tests of liver (ALT and AST) and kidney (UA and BUN) function. All experiments were repeated three times. All data are expressed as the mean ± SD. One-way ANOVA and Tukey's multiple comparisons test were used for statistical analysis. *p < 0.05, **p < 0.01, ***p < 0.001. ALT, alanine aminotransferase; AST, aspartate transaminase; UA, uric acid; BUN, blood urea nitrogen; HE, hematoxylin and eosin.

We evaluated the mechanical properties, swelling properties, morphology, degradation properties, and release kinetics of hydrogels. The hydrogel precursors did not cross-link at 4°C and remained in a sol state, indicating that they are injectable (Figure 10A). H1 exhibited superior swelling performance (Figure 10B), greater compressive strength (Figure 10C), a more robust stress-strain curve (Figure 10D), and a more appropriate gelation time (Figure 10E) compared with those of H2 and H3. As depicted in Figure S7A-C, the 3D network structure formed by H1 was both uniform and dense, contributing to improved swelling and mechanical properties. This is because the formation of CS and β-GP networks in H1 is more conducive to structural stability (FTIR demonstrated the formation of hydrogen bonds and complexes between CS molecular chains and β-GP). H2 showed a lower compressive strength than that of H1-β-GP. However, due to the further decrease in the overall concentration of H3, the porosity of the three-dimensional network negatively impacts compressive performance. H3, with its lower CS content in the organic matrix, forms a less effective network, resulting in diminished swelling capabilities. Furthermore, the degradation cycle of the H1 hydrogel was extended, allowing for a sustained effect over a longer duration (Figure 10F). As shown in Figure 10G, the virus continued to be released for nearly 4 weeks *in vitro*. These results demonstrate the feasibility of virus-controlled release in hydrogels as a whole. The stable and sustained degradation rate of the hydrogel in this environment facilitates the sustained release of the virus. Moreover, as shown in Figure 10H, cross-linking of hydrogels decreased with increasing shear stress. An SEM image analysis of the lyophilized hydrogel (Figure 10I) indicated that the hydrogel has a smooth microstructure and uniform wall thickness as well as interpenetrating pores with diameters of approximately 10 μm. On the basis of the negative charge of viruses and positive charge of CS, virus particles with a diameter of approximately 100 nm were embedded in the hydrogel. When the hydrogel was rapidly heated from 4℃ to 37℃, Figure 10E and J show that gelation began in 60 s, indicating gel formation at body temperature. Additionally, the hemolysis rate caused by H1 was less than 1%, lower than that caused by Triton (Figure 10K). The live/dead cell staining results on days 1, 3, and 6 are shown in Figure 10L. There were almost no inactivated cells in each group and no significant differences in the proportion of live/dead cells among all groups (p > 0.05). The CCK8 assay results were consistent with this observation (Figure S7D). To further investigate the potential visceral toxicity, comprehensive histological assessments were performed on tissue sections from the heart, liver, spleen, lungs, and kidneys. As depicted in Figure 10M, there were no substantial pathological changes within these organs. Furthermore, the liver and kidney functions remained unaltered, with no significant deviations observed in corresponding functional markers, as shown in Figure 10N-Q.

### Animal research

**Figure 11:**
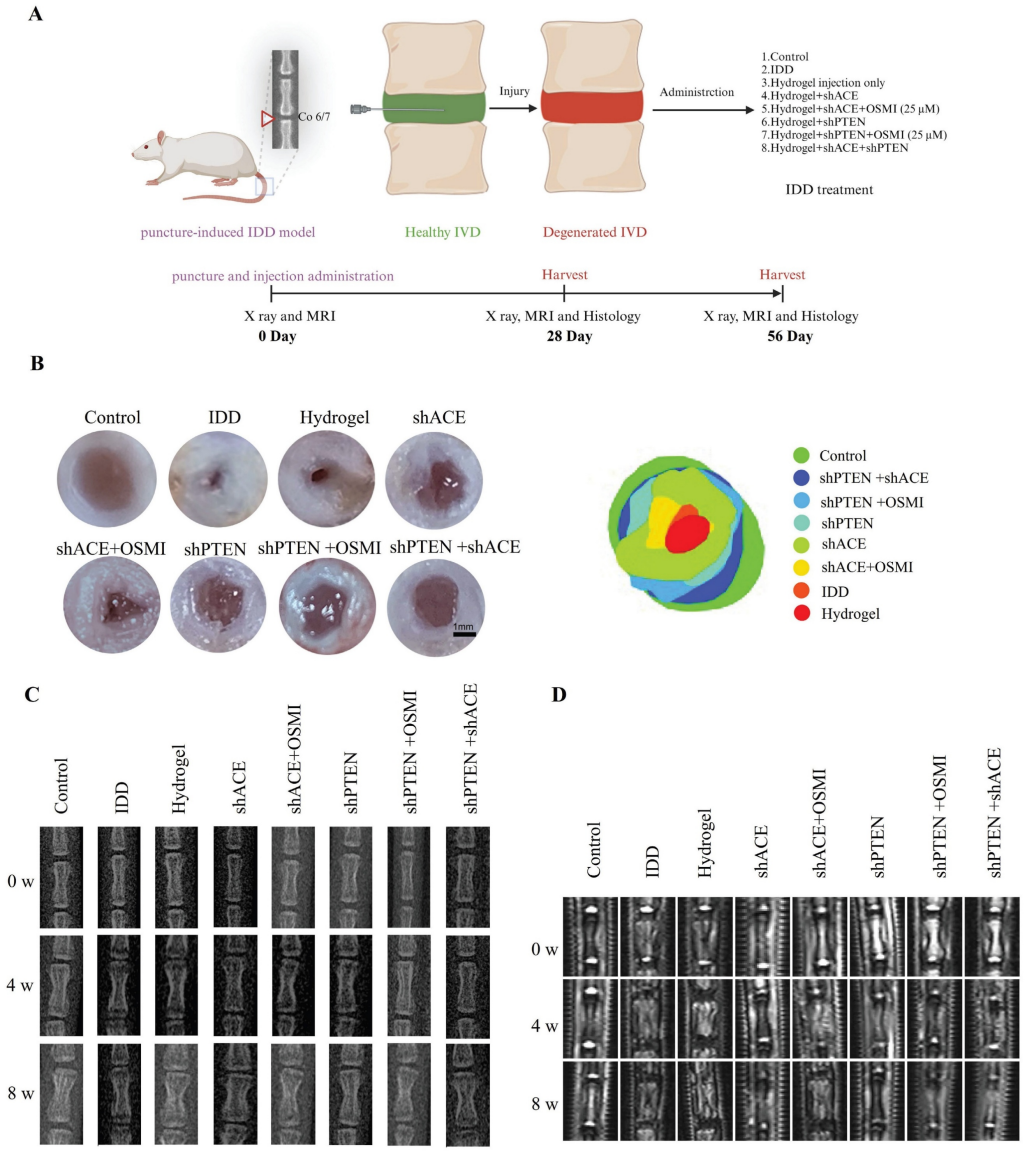
Macroscopic observation of IVD and radiographic evaluations of rat tail. A: Schematic diagram of animal experiment. B: Macroscopic IVD images, fitting picture, and the relative area of nucleus pulposus at 8 weeks after the intervention. C: Representative X ray radiographs of the rat tail at 0, 4 and 8 weeks after the intervention. D: Representative MRI scans were obtained at 0, 4 and 8 weeks after the intervention. All experiments were repeated ten times. IVD, intervertebral disc; MRI, magnetic resonance imaging.

A schematic of the animal model experiments is shown in Figure 11A. There was a strong correlation between the general morphology of IVD and the IDD and Pfirrmann grades. As shown in Figure 11B, both the control and hydrogel+shACE+shPTEN groups displayed a typical IVD morphology, with a high-water content and clear boundaries (NP and AF). The NP volume in IVD decreased in the hydrogel+shACE, hydrogel+shPTEN, and hydrogel+shPTEN+OSMI groups. The IDD, hydrogel, and hydrogel+shACE+OSMI groups showed a disordered IVD tissue structure, decreased NP volume, and thickened fibers. There were significant differences between the hydrogel+shACE+shPTEN group and other groups with respect to the relative area of NP, except in comparison with the control group.

X-rays and MRIs (T2-weighted imaging) were used to assess the severity of IDD. As shown in Figure 11C and Figure S8A-C, the DHI%, an important metric for assessing the severity of IDD, in other groups was lower than those in the control, hydrogel+shPTEN, and hydrogel+shACE+shPTEN groups 0 weeks after implantation (p < 0.05), with no significant differences among other groups. All groups except the control group showed a decrease in DHI% at 4 weeks after implantation. DHI% was significantly higher in hydrogel+shACE, hydrogel+shPTEN, hydrogel+shPTEN+OSMI, and hydrogel+shACE+shPTEN groups than in the IDD, hydrogel, and hydrogel+shACE+OSMI groups, and ACE and PTEN transfection groups were more effective than single virus transfection groups. DHI% was expected to decline rapidly in the IDD group. While the hydrogel+shACE, hydrogel+shPTEN, hydrogel+shPTEN+OSMI, and hydrogel+shACE+shPTEN groups showed some degree of IVD compression, DHI% was still high in the hydrogel+shACE+shPTEN group. The hydrogel for virus delivery was capable of maintaining the IVD height to some extent.

MRI is currently the gold standard for evaluating IDD and is capable of detecting the water content in IVD. As shown in Figure 11D and Figure S8D-F, MRI indicators at week 0 after implantation were consistent with X-ray results. At 4 weeks after implantation, MRI-T2 signals decreased in all groups except the control group. MRI T2 signals declined more rapidly in the IDD, hydrogel, and hydrogel+shACE+OSMI groups than in the hydrogel+shACE, hydrogel+shPTEN+OSMI, hydrogel+shPTEN, and hydrogel+shACE+shPTEN groups (p < 0.05). Approximately 8 weeks after implantation, except for the control group, the T2 signal decreased severely in the IDD, hydrogel, and hydrogel+shACE+OSMI groups, while it decreased moderately in the hydrogel+shACE, hydrogel+shPTEN, and hydrogel+shPTEN+OSMI groups and showed a slight decrease in the hydrogel+shACE+shPTEN group.

**Figure 12:**
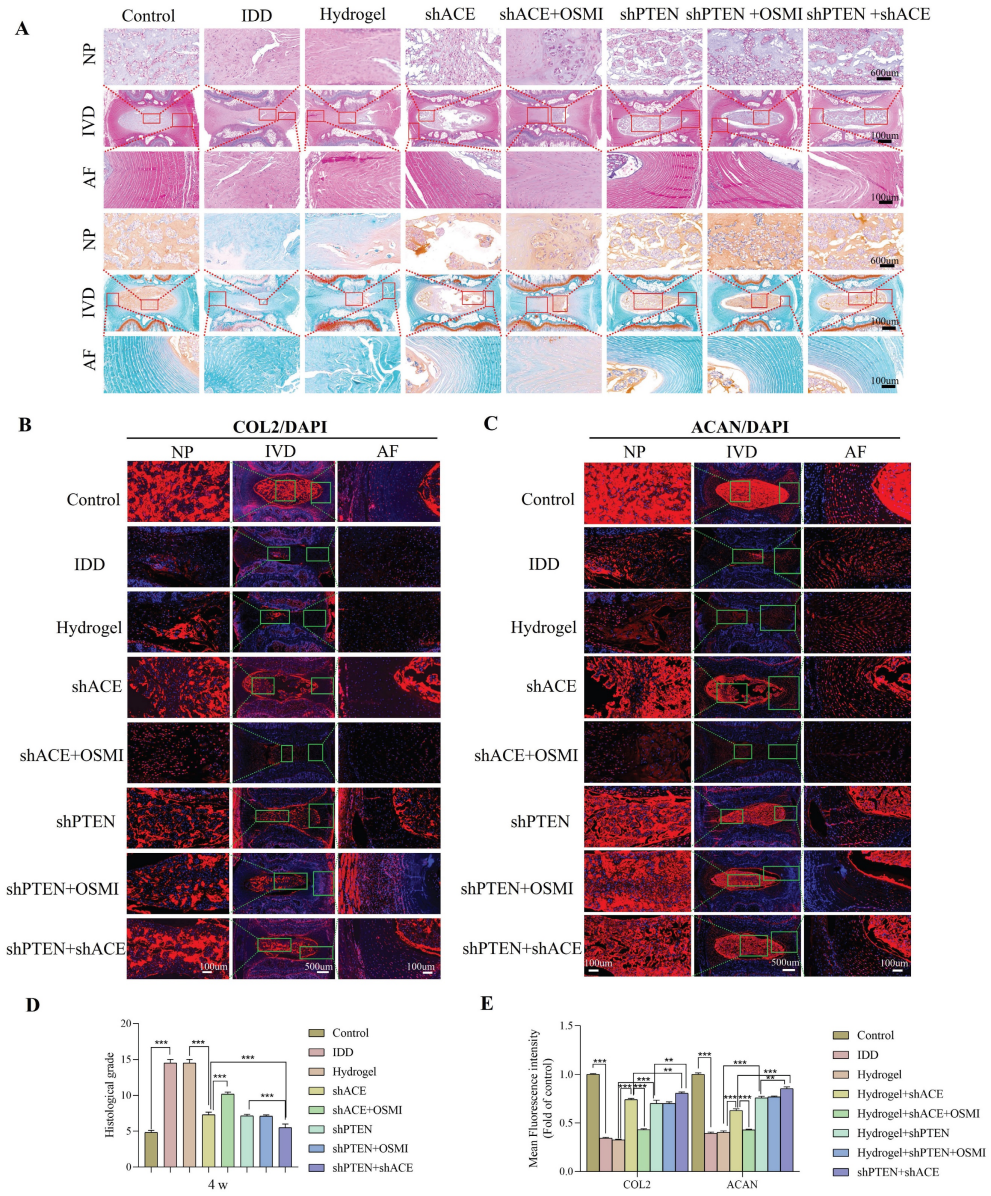
Effects of composite hydrogel loaded with the virus on histological scores and extracellular matrix corresponding to rat coccygeal IVDs at 4 weeks after the intervention. A: Representative HE staining and SO staining images of rat coccygeal IVDs corresponding to different treatment groups (coronal position). B-E: Immunofluorescence detection of COL2 and ACAN at 4 weeks after the intervention and the quantitative analysis of immunofluorescence staining. All experiments were repeated ten times. All data are expressed as the mean ± SD. One-way ANOVA and Tukey's multiple comparisons test were used for statistical analysis. *p < 0.05, **p < 0.01, ***p < 0.001. IVD, intervertebral disc; HE, hematoxylin and eosin. SO, safranin-o/fast green; COL2, Collagen II; ACAN, aggrecan.

**Figure 13:**
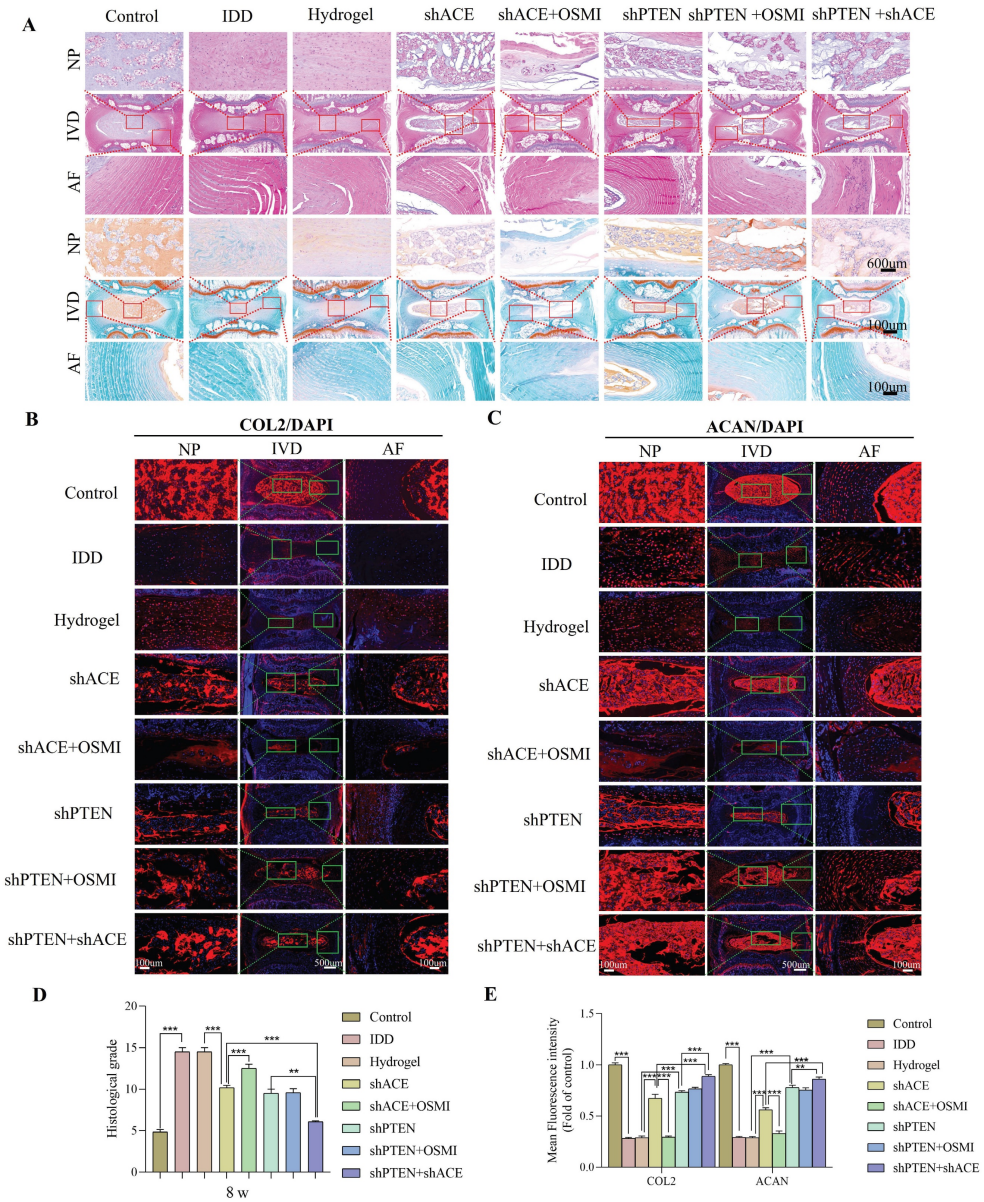
Effects of composite hydrogel loaded with the virus on histological scores and extracellular matrix corresponding to rat coccygeal IVDs at 8 weeks after the intervention. A: Representative HE staining and SO staining images of rat coccygeal IVDs corresponding to different treatment groups (coronal position). B-E: Immunofluorescence detection of COL2 and ACAN at 8 weeks after the intervention and the quantitative analysis of immunofluorescence staining. All experiments were repeated ten times. All data are expressed as the mean ± SD. One-way ANOVA and Tukey's multiple comparisons test were used for statistical analysis. *p < 0.05, **p < 0.01, ***p < 0.001. IVD, intervertebral disc; HE, hematoxylin and eosin. SO, safranin-o/fast green; COL2, Collagen II; ACAN, aggrecan.

As shown in Figure 12A and Figure 13A, HE and SO staining revealed that NP in the control group was well organized, whereas IVD in the IDD, hydrogel, and hydrogel+shACE+OSMI groups was disorganized or even missing. Following implantation of hydrogel+shACE, hydrogel+shPTEN, and hydrogel+shPTEN+OSMI materials, the IVD tissue content was reduced, AF was disordered, and a small amount of NP tissue was retained. Although the IVD in the hydrogel+shACE+shPTEN group was degraded to some extent, the quantity and structure of NP and AF in the hydrogel+shACE and hydrogel+shPTEN groups were superior to those in the hydrogel+shACE and hydrogel+shPTEN groups. At 4 and 8 weeks after implantation, histological scores in other groups were significantly higher than those in the control group (p < 0.05). The hydrogel+shACE, hydrogel+shPTEN, and hydrogel+shPTEN+OSMI groups had significantly lower mean scores than those of the IDD, hydrogel and hydrogel+shACE+OSMI groups 8 weeks following implantation (p < 0.05). The histological scores for the hydrogel+shACE+shPTEN group were significantly lower at 4 and 8 weeks than those for the hydrogel+shACE, hydrogel+shPTEN, and hydrogel+shPTEN+OSMI groups (p < 0.05). After 4 and 8 weeks of implantation, IF results (Figure 12B-E, Figure 13B-E) indicated that, except for the control group, the expression levels of COL2 and ACAN in the hydrogel+shACE, hydrogel+shPTEN+OSMI, and hydrogel+shACE+shPTEN groups were significantly higher than those in other groups (p < 0.05). Furthermore, the analysis revealed statistically significant differences between the hydrogel+shACE+shPTEN group and the hydrogel+shACE or hydrogel+shPTEN groups as well as between the hydrogel+shACE and hydrogel+shACE+OSMI groups.

## Discussion

In addition to being an endocrine system within the circulatory system, the RAAS functions in many local tissues 25. The physiological and pathological functions of many tissues and organs are adversely affected by circulatory or local RAAS abnormalities 26. In this study, we found that ACE knockdown improves the aging and degeneration of NP cells. Moreover, we found that PTEN regulates K48-linked ubiquitination of ACE mediated by TRIM63, and PTEN knockdown improves NP degeneration and aging phenotypes. In the ubiquitination degradation of ACE regulated by PTEN, we found that PTEN-induced dephosphorylation of TRIM63 and PLK1-induced phosphorylation of TRIM63 were crucial for the ACE-TRIM63 interaction. These findings provide new insights into the mechanisms by which ACE and TRIM63 are regulated. Furthermore, a decrease in ACE protein levels reversed the up-regulation of PTEN, thus forming a negative feedback mechanism and affecting the above molecular interactions. This feedback loop also influenced ER autophagy through the regulation of *O*-GlcNAc modification, thus playing a significant role in the regulation of IDD. In particular, ACE knockdown attenuated the adverse effects of ER autophagy on NP cells by regulating glycosylation modification levels, and similar effects were not observed for PTEN, as demonstrated both *in vivo* and *in vitro*. These findings provide a new understanding of the construction and regulation of the PTEN-ACE axis.

In addition to its role as a principal effector of conventional RAAS, Ang II serves as a potent pro-inflammatory mediator that affects the nuclear translocation of the p65 subunit, transcription of NF-κB, and degradation of the transcription inhibitor IκB 27, 28. The activation of Ang II/AT1R not only results in vasoconstriction, inflammation, and organ fibrosis but also increases the production of adhesion molecules and chemokines 29, 30. Angiotensin-converting enzyme 2 (ACE2) catalyzes the conversion of Ang II into Ang-1-7 31. Mas receptor (MasR) is a major Ang-(1-7) receptor 32. The anti-apoptotic, vasodilator, anti-inflammatory, anti-hypertrophic, and antifibrotic properties of Ang-(1-7) are enhanced when combined with MasR 33, and this is related to the inhibition of Ang II/AT1R. The RAAS is frequently activated during both acute and chronic organ injury, whereas the ACE2/Ang-(1-7)/MasR axis is down-regulated and the ACE/Ang II/AT1R axis is up-regulated. This results in an imbalance between ACE2 and ACE as well as between Ang II and Ang-(1-7), ultimately causing endothelial dysfunction and heightened oxidative stress 34. In addition to contributing to immune cell infiltration, RAAS contributes to inflammation and fibrosis associated with kidney ischemia and myocardial infarction 35, 36. In particular, macrophage polarization from the M2 anti-inflammatory subtype to the M1 pro-inflammatory subtype perpetuates inflammation and tissue remodeling. In general, RAAS promotes inflammation, influences adipocyte differentiation, and regulates adipokines, thereby contributing to metabolic disease 37. Therefore, these pathophysiological processes could be altered by modulating RAAS activation. As an example, lipid receptor agonists may reduce lipopolysaccharides-induced lung and liver damage through RAAS modulation 38. In addition, it has recently been demonstrated that ACE inhibitors (ACEIs) reduce pancreatic inflammation and fibrosis in patients with chronic pancreatitis 39. There is no evidence for an association between human autoimmunity and ACEI therapy to date; however, this treatment improves outcomes for some autoimmune diseases, including rheumatoid arthritis, multiple sclerosis, and lupus, by reducing pro-inflammatory cytokines 40, 41.

ACE, a zinc-dependent dipeptidyl carboxypeptidase, is a crucial synthase of Ang II, a significant effector in the RAAS. ACE and Ang II have been investigated concerning vascular inflammation and injury; their inhibition results in diminished inflammation, reduced recruitment of leukocytes, and less damage to vascular walls, which are critical characteristics of chronic inflammatory and autoimmune disorders, including renal tubulointerstitial injury and diabetic nephropathy 42. Excessive involvement of ACE/Ang II in the brain causes oxidative stress, neuroinflammation, and apoptosis, resulting in various neurological disorders 43. Increased serum ACE levels are regarded as an inflammatory sign for diagnosing sarcoidosis, an autoimmune inflammatory disorder 44. Increased ACE activity has been noted in neointima following experimental artery injury, and a rise in ACE expression is associated with an elevated risk of myocardial infarction 45. Consequently, ACE inhibition decreases arterial NF-κB activation in macrophages and vascular smooth muscle cells, which are pivotal in the pathophysiology of atherosclerosis 46. The activation of NF-κB is predominantly linked to inflammatory processes, and the identification of NF-κB as an endothelial ACE transcriptional activator may establish an additional connection between ACE and the onset of inflammation 47. Moreover, ACE is associated with immunological modulation and has a role in antigen presentation 48. Endogenous ACE has been identified in a variety of immune cell types, including macrophages and neutrophils. Its overexpression leads to an enhanced antitumor response, antigen presentation, and bactericidal activity, independently of Ang II 49. ACE^10/10^ model mice, which exhibit 16 to 25 times higher ACE expression than that in wild-type mice, show an augmented monocyte and macrophage immune response characterized by a heightened pro-inflammatory phenotype of macrophages, an intensified inflammatory response, and increased aggressiveness 50. Given the role of ACE in peptide processing for antigen presentation, its overexpression likely augments antigen presentation and activates immune pathways rather than establishing a distinct microenvironment. ACE overexpression in neutrophils increases bactericidal and oxidative responses, evidenced by increased superoxide production (including phagocytosis) and the formation of neutrophil extracellular traps, highlighting the direct correlation between bacterial eradication and ACE production 51. In summary, ACE can modulate inflammation, oxidative stress, and immunological responses, and its overexpression is linked to apoptosis, inflammation, and fibrosis. Consistent with these functions, in this investigation, the degeneration and senescence of NP cells were ameliorated by ACE knockdown. Furthermore, ACE may function via modulating the phosphorylation activation of PI3K/Akt/mTOR. Therefore, we evaluated upstream regulators of ACE and the PI3K/Akt pathway. We focused on the PI3K/Akt regulator PTEN, which affects overall levels of Ub and proteasome function, suggesting that it is involved in the regulation of ACE proteasome degradation 52, 53. qPCR and WB analyses revealed that PTEN influences ACE expression solely at the protein level, without affecting the RNA level. Additionally, we identified E3 ubiquitin ligases associated with ACE. Previous research as indicated that sarcopenia is associated with the pathophysiology of IDD 23. The reduction in skeletal muscle mass may contribute to the development of IDD through two primary mechanisms: altering spinal biomechanical properties and influencing the local inflammatory microenvironment. TRIM63, functioning as an E3 ubiquitin ligase, facilitates the ubiquitination and subsequent proteasomal degradation of many proteins, including troponin I and peroxisome proliferator-activated receptor alpha (PPARα), via a ring finger-dependent process 54-56. Of note, the results of this study indicated that TRIM63, a crucial regulator of sarcopenia, is linked to skeletal muscle atrophy but also to the pathological process of IDD by regulating ACE ubiquitination degradation. This similarity indicates that TRIM63 could serve as an important molecular bridge connecting sarcopenia and IDD. On the basis of these findings, we hypothesize that the TRIM63-ACE regulatory axis may represent an important pathological mechanism in sarcopenia-associated IDD, a hypothesis that requires verification through further mechanistic studies.

PTEN acts as both a lipid phosphatase and a protein phosphatase, dephosphorylating protein substrates at tyrosine, serine, and threonine residues, while also converting lipid phosphatidylinositol 3,4,5-triphosphate into phosphatidylinositol 4,5-diphosphate, thus regulating cell cycle progression and cell survival. PTEN diminishes the activity of NP cells, promotes death and senescence, and decreases the expression and synthesis of ECM components 57. As expected, in this study, NP cell degeneration and senescence were ameliorated following PTEN knockdown. PTEN dephosphorylates several substrates and contributes to various cellular functions, including the inhibition of cell adhesion and migration as well as the preservation of genomic integrity 58. Furthermore, PTEN exhibits multiple biological functions, such as the induction of apoptosis and G1 phase cell cycle arrest, prevention of cell survival, motility, adhesion, and migration, along with the maintenance of genomic integrity 59. A non-phosphorylated variant of PTEN is destabilized via the ubiquitin-proteasome pathway 16. A prior study has indicated that appropriate phosphorylation of PTEN is crucial for safeguarding the PTEN protein against proteasomal destruction 12. The phosphorylated C-terminal tail conceals the degradation signal in the PTEN protein, and dephosphorylation can expose this signal, resulting in the swift degradation of PTEN. Phosphorylation of PTEN at Thr366, Ser370, and C-tail locations, including Ser380, Thr382, Thr383, and Ser385, facilitates the interaction between the C-tail and N-terminal C2 and phosphatase domains. The phosphorylation-induced conformational shift of PTEN to a "closed" state correlates with PTEN inactivation and enhanced protein stability. The alteration of the pertinent phosphorylation site to non-phosphorylated alanine prompts a conformational shift in PTEN to an "open" state, hence augmenting its membrane affinity and enzymatic activity, resulting in heightened instability and expedited breakdown of PTEN. The typical biological function of PTEN is regulated by the equilibrium between its phosphorylation and dephosphorylation. Phosphorylation at Ser380-Ser385 enhanced the half-life of PTEN while significantly diminishing its enzymatic activity. Conversely, Thr366 phosphorylation led to diminished PTEN stability and adversely affected the capacity of PTEN to inhibit cell invasion. PTEN phosphorylation influences cellular localization because the introduction of phosphorylation-mimicking mutations in the Ser380-Ser385 cluster results in less membrane binding. Thr383 phosphorylation also impeded the capacity of PTEN to control cell migration, indicating that phosphorylation of Ser380-Ser385 typically has adverse effects of the functions of PTEN. Furthermore, the majority of PTEN point mutations occur within its phosphatase domain, notably C124S, G129E, Y138L, and R130G alterations, leading to a loss of PTEN phosphatase activity 11. We generated a PTEN C124S enzyme-inactivated mutant and subsequently co-transfected Flag-TRIM63 into NP cells, observing that the phosphorylation level of TRIM63 exceeded that of wild-type PTEN. Furthermore, transfection with mutant plasmids revealed that His-PTEN S380A, His-PTEN T382A, and His-PTEN T383A influence the enzymatic activity of PTEN. This study primarily focused on the dephosphorylation of substrates. To further assess the impact of the three phosphorylation sites on PTEN or phosphatase activity, we evaluated the phosphorylation levels of TRIM63 following the transfection of mutated plasmids at these phosphorylation sites (His-PTEN S380A, T382A, and T383A). Transfection with the first two plasmids reduced the phosphorylation level of TRIM63, whereas transfection with the His-PTEN T383A plasmid resulted in a comparable phosphorylation level to that of the WT (Figure 8D). Phosphatase activity increased for the first two PTEN variants and was not significantly altered for the third. In other words, as the phosphorylation of PTEN decreased due to mutations at the first two phosphorylation sites, the phosphorylation of TRIM63 also increased. This, in turn, slowed down the ubiquitination-mediated degradation rate of ACE, which aligns with previous findings. However, the phosphorylation of Thr383 showed a different trend, inconsistent with prior research, warranting further investigation. Interestingly, in this study, we observed that while PTEN expression levels increased with degeneration, its phosphorylation levels decreased, leading to its destabilization. This finding may partially explain why PTEN, despite its significant regulatory role, does not always directly contribute to the development of certain diseases. It also highlights the complexity of the underlying mechanisms. Subsequently, we performed CHX tracking tests, showing that the decay rate of the ACE protein was attenuated following transfection with His-PTEN S380A and His-PTEN T382A mutant plasmids but not His-PTEN T383A. Following ACE knockdown, PTEN protein and phosphorylation levels increased, resulting in elevated phosphorylation levels of S380 and T382. This, in turn, diminished the phosphatase activity of PTEM and heightened the phosphorylation level of TRIM63, ultimately enhancing the ubiquitin-proteasome degradation of ACE. Nonetheless, the elevation of PTEN levels resulted in a modest rise in ACE protein levels; however, it was unable to fully counteract the effects of ACE deletion. These data indicate that the knockdown of both PTEN and ACE can enhance NP cell degeneration and senescence, with a more pronounced degree of improvement when both are knocked down simultaneously (Figure S3).

The ER is one of the most versatile and adaptable organelles in eukaryotic cells and is responsible for controlling the synthesis, folding, and maturation of luminal, secretory, and transmembrane proteins. ER stress generates a multifaceted array of cellular signals capable of modifying gene expression, cellular biochemistry, and signaling networks, thereby contributing to the pathogenesis of various metabolic disorders, including nonalcoholic fatty liver disease, diabetes, and fibromyalgia 60, 61. By activating pro-apoptotic pathways and inducing epithelial-mesenchymal transformation, ER stress promotes fibrotic remodeling 62. A number of studies have demonstrated that ER stress can induce inflammatory responses by activating the MAPK/JNK pathway as well as the NLRP3 inflammasome, which regulates the maturation of IL-1β and IL-18 63, 64. Glycosylation involves glycans (monosaccharides or oligosaccharides) attaching to proteins in the ER and Golgi bodies that serve as quality control signals during the folding of ER proteins. Thus, glycosylation plays an important role in the regulation of ER metabolism and protein quality. The dysregulation of protein glycosylation/deglycosylation leads to glycoproteins being trapped in the ER, thus increasing the risk of ER stress. During ischemic conditions, glycosylation of newly synthesized proteins is impaired, resulting in ER accumulation and excess ER stress, thereby activating caspase12^65^. Typically, tunicamycin inhibits glycosyltransferases by blocking N-linked glycosylation, resulting in the disruption of protein maturation along with the induction of apoptosis and ER stress 66. Studies have shown that O-glycosylation inhibitors can also induce apoptosis and prevent the invasion of cancer cells 67. Similarly, 2-deoxy-d-glucose (2-DG), a glucose analogue, inhibits protein glycosylation and induces misfolded proteins to accumulate within the ER, leading to prolonged apoptosis 68. In a subsequent study, it was noted that OSMI-1 induced an increase in the autophagy-associated protein LC3B, decrease in p62, and significant increase in autophagosomes in shACE-transfected NP cells. ACE, a key downstream molecule of PTEN, may regulate ER stress-related signaling pathways to influence the initiation of ER autophagy 69. It may modulate the accumulation of misfolded proteins to activate or inhibit the autophagy process. In addition, PTEN may indirectly regulate the expression and function of autophagy-related proteins (such as LC3 and p62) through the PI3K/AKT/mTOR pathway 70. Additionally, the PTEN-ACE pathway may influence the recognition and degradation of ER fragments by regulating the expression or function of selective ER autophagy receptors (such as SEC62) 71. Finally, the PTEN-ACE pathway may also affect the synergistic effects of ER autophagy and mitochondrial autophagy by regulating the function of ER-mitochondria contact sites 72. It may indirectly regulate ER autophagy by modulating calcium signaling or lipid metabolism. *O*-GlcNAc glycosylation modified key proteins involved in autophagy with both inhibitory and stimulatory effects 73, 74. Studies of IDD models have indicated that *O*-GlcNAc glycosylation affects the abundance of the 134 member B (FAM134B) protein in the family with sequence similarity 134, which in turn affects autophagy within the ER 75. As in the acute stress model, OGT overexpression inhibited apoptotic marker expression (such as caspase 3) in IDD. A lack of glycosylation sites 158 or 169 on hemagglutinin (HA) increases influenza virus virulence by activating strong ER stress and inflammation, which contribute to disease progression 76. Therefore, correct glycosylation is essential to the half-life of circulating proteins, their affinity for receptors, and their antigenicity.

ACE may regulate overall *O*-GlcNAc modification levels in cells by modulating the expression or activity of OGT. Furthermore, RAAS, including ACE, may affect hexosamine biosynthetic pathway flux by regulating glucose metabolism, glutamine metabolism, or fatty acid metabolism, thereby altering the production of uridine diphosphate *N*-acetylglucosamine (UDP-GlcNAc) and the level of *O*-GlcNAc modification 77. Finally, *O*-GlcNAc modification may regulate the stability, activity, or subcellular localization of ACE itself through feedback loops (e.g., by influencing the ubiquitination-mediated degradation rate of ACE or its interactions with other proteins). ACE-overexpressing macrophages and neutrophils have higher levels of TCA cycle intermediates, which are used by other immune cells to compensate for bursts of glycolysis and signaling molecules 78. Furthermore, the increase in TCA cycle intermediates suggests that macrophages and neutrophils use them as precursor molecules for other bactericidal products to enhance phagocytosis 79, 80. ATF4/CHOP/GRP78 axis has been reported to induce podocyte ERS via Ang II; RAAS activation can be altered to reduce this effect 81. Valsartan, for example, improves tubular morphology and relieves ER stress in proximal renal tubules, confirming the role of local RAAS in renal structural and functional impairment under high-fat diets 82. Conversely, inhibiting RAAS with valsartan did not attenuate Tunicamycin-induced endoplasmic reticulum stress, a standard chemical inducer, indicating that RAAS activation is specific to Tunicamycin-induced endoplasmic reticulum stress in proximal renal tubule cells; however, the underlying mechanism is unclear. It should be noted that ACE2, which is counterbalanced with ACE, improves skeletal muscle lipid metabolism and ER stress in C2C12 cells 83. It has also been demonstrated that ACE2 alleviates ERS-induced hepatic steatosis and insulin resistance via IKKβ/NF-κB/IRS1/Akt 84. A detailed analysis of the role of ACE2 in the prevention of insulin resistance caused by ER stress has indicated that it inhibits gluconeogenesis and stimulates glycogen synthesis 85. Although no studies have determined whether ACE can regulate ERS, ACE and ACE2 are antagonistic and may share some functional similarities. We found that ACE knockdown substantially reduced the increase in autophagy protein LC3B and the decrease in p62 caused by IL-1, and TEM results indicated that this improvement had no significant impact on the number of autophagosomes or the structure of the ER.

We leveraged the positive charge of CS for simple and rapid viral loading through strong electrostatic interactions. Additionally, we introduced β-GP to develop a temperature-sensitive hydrogel material with a modulus that matches that of human soft tissue. The hydrogel forms a compact network within 30 s at body temperature, facilitated by enhanced hydrogen bonding among CS molecules. By adjusting hydrogel formulation, we achieved precise control over the network density, enabling tunable degradation rates and controlled viral release in target tissues. Notably, TEM images clearly demonstrated the structural integrity of virus particles loaded onto the CS hydrogel. Subsequent *in vivo* experiments confirmed that the virus loading and release capabilities of the CS hydrogel enable robust gene knockdown. While this study demonstrates the ability to adjust and control the virus loading and degradation rates of temperature-sensitive CS hydrogels, it is important to note that CS properties, such as its molecular weight and deacetylation degree, can vary between batches due to differences in production. Therefore, the relationships between the formulation, network density, degradation rate, and virus release rate must be carefully considered when using CS hydrogels from different batches. Additionally, the current system lacks tissue specificity, which may result in non-targeted virus release. Addressing these challenges and advancing the practical application of this delivery system will require interdisciplinary collaboration, involving molecular modification, process optimization, and rigorous quality control.

## Supplementary Material

Supplementary figures and tables.
