# Supplementary material for: Exploring Negative Feedback Mechanisms in the PTEN-ACE Axis: Application of Electrosorb Hydrogel-Based Gene Delivery for Intervertebral Disc Regeneration
Source: Int J Biol Sci. 2025 May 15;21(8):3416–43. doi: 10.7150/ijbs.111898 (PMC12160554; doi:10.7150/ijbs.111898)
Supplement: Supplementary file 1 — Supplementary figures and tables. [file ijbsv21p3416s1.pdf]

Table S1. Clinical information of human degenerative disc samples from 9 patients.

Variables	Patient 1	Patient 2	Patient 3	Patient 4	Patient 5	Patient 6	Patient 7	Patient 8	Patient 9
Sex	Male	Female	Male	Male	Female	Male	Male	Female	Male
Age	26	35	45	47	56	52	63	67	75
Diagnosis	LDH	LDH	LDH&LSS	LDH&LSS	LDH&LSS	LDH&LSS	LDH&LSS	LDH&LSS	LDH&LSS
Level	L4/5	L4/5	L5/S1	L5/S1	L5/S1	L4/5	L5/S1	L4/5	L4/5
Grades	II	II	II	III	III	III	V	V	V

LDH, lumbar disc degeneration; LSS, lumbar spinal stenosis.

Table S2. Antibodies used in this study

Antibody	Manufacturer	Catalog No.
COL2	Proteintech	28459-1-AP
p21	Proteintech	28248-1-AP
P16	Proteintech	10883-1-AP
ADAMTS4	Proteintech	11865-1-AP
PI3K	Abmart	T40115
p-PI3K	Abmart	T40116
Akt	Abmart	T55561
p-Akt	Abmart	T40067S
mTOR	Proteintech	66888-1-Ig
p-mTOR	Proteintech	67778-1-Ig
ACE	Proteintech	24743-1-AP
PTEN	Proteintech	22034-1-AP
TRIM63	Abclonal	A3101
TRIM63	Santa Cruz Biotechnology	sc-398608
OGT	Proteintech	66823-1-Ig
ubiquitin	Proteintech	80992-1-RR
O-GlcNAc	Abcam	ab2739
p62	Proteintech	80294-1-RR
LC3B	Abclonal	A19665
p-PTEN	Abclonal	AP1346
PLK1	Abclonal	A21082
β-actin	Abclonal	AC026
GAPDH	Proteintech	60004-1-Ig
Flag	Cell Signaling Technology	14793
Myc	Cell Signaling Technology	2276
HA	Cell Signaling Technology	3724
p-S/T	BD Biosciences	612549
His	Cell Signaling Technology	12689

COL2, Collagen II; ACAN, aggrecan; ADAMTS4, a disintegrin and metallo-proteinase with thrombospondin motif 4; ACE, angiotensin converting enzyme; PTEN, phosphatase and tensin homolog; PI3K/Akt/mTOR, phosphoinositide 3-kinase/protein kinase B/mammalian target of rapamycin; PLK1, polo-like kinase 1; TRIM63, tripartite motif containing 63; OGT, O-linked N-acetylglucosamine (GlcNAc) transferase.

Table S3. Primer sequences for reverse transcription–polymerase chain reaction.

	Forward primers	Reverse primers
COL2	5′-CAAGAACAGCATTGCCTATCTG-3′	5′-GATAACAGTCTTGCCCCACTTA-3′
MMP13	5′-AGACCCCAACCCTAAACATCC-3′	5′-AAAACAGCTCCGCATCAACC-3′
P16	5′-CACTTCTCACCTCGCTTGTCACA-3′	5′-CCAAGAACCTGCGACCCATGCT-3′
P21	5′-GCCAGATTTGTGGCTCACTTCG-3′	5′-ACGCTTGGCTCGGCTCTGG-3′
ACE	5′- GCCCTGCAGGTGTCTGCAGCATGT-3′	5′-GGATGGCTCTCCCCGCCTTGTCTC-3′
PTEN	5′-CTGCAGAAAGACTTGAAGGCG-3′	5′-GGGAATAGTTACTCCCTTTTTGTC-3′
GAPDH	5′-AGCCACATCGCTCAGACAC-3′	5′-GCCCAATACGACCAAATCC-3′

COL2, collagen type II; MMP13, matrix metalloproteinase 13; ACE, angiotensin-converting enzyme; PTEN, phosphatase and tensin homolog.

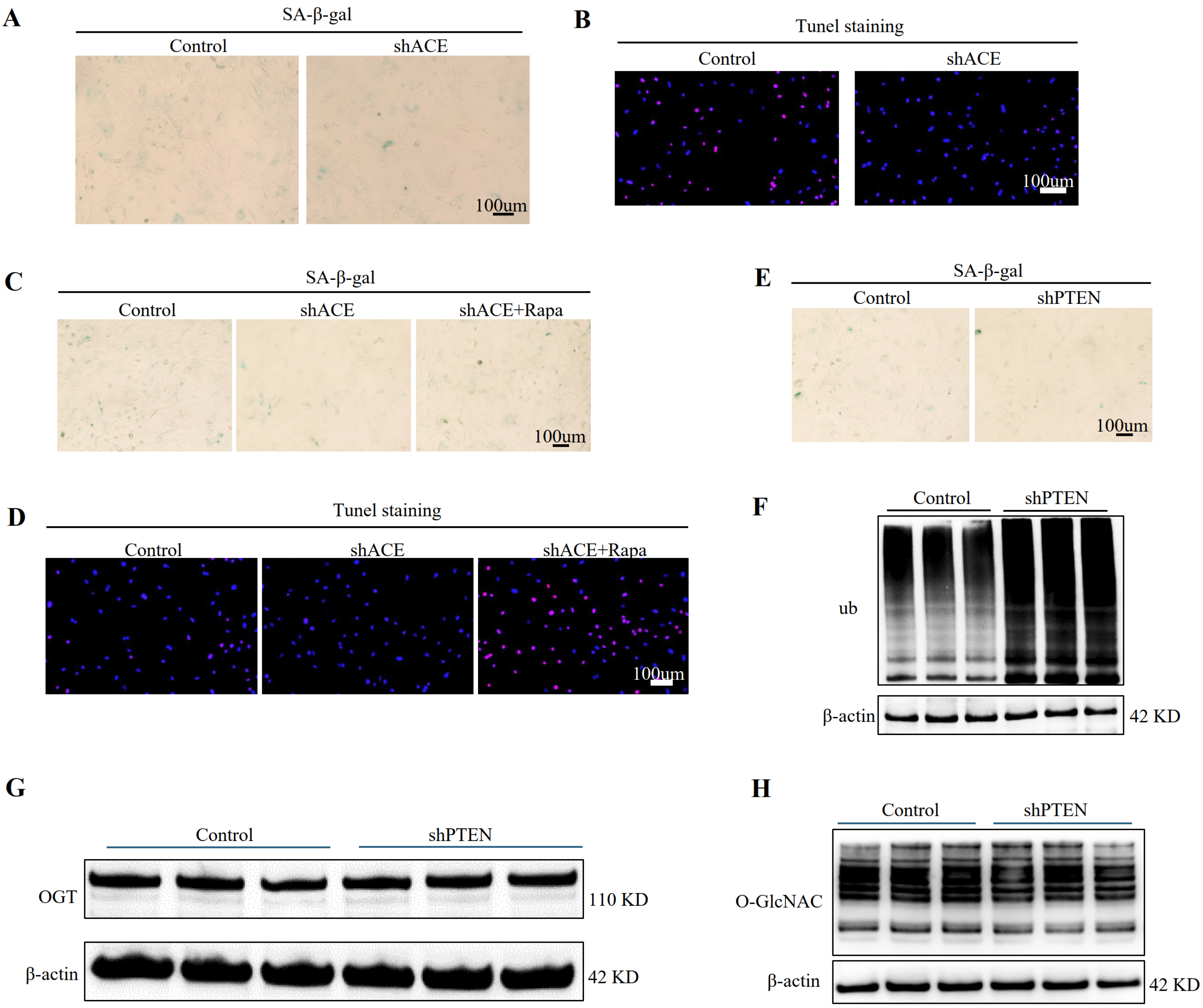

Figure S1 The functional verification of ACE and PTEN. A and B: Representative images of TUNNEL and β-galactosidase staining of NP cells transfected with shACE. Scale bar: 100 µm. C and D: Representative images of TUNNEL and β-galactosidase staining of NP cells transfected with shACE along with the mTOR inhibitor (rapamycin, Rapa, 100 nM) or not. Scale bar: 100 µm. E: Representative images of β-galactosidase staining of NP cells transfected with shPTEN. F, G and H: The proteins ubiquitin (Ub), OGT, and O-GlcNAc levels of NP cells transfected with shPTEN. All experiments were repeated three times. ACE, angiotensin converting enzyme; PTEN, phosphatase and tensin homolog; NP, nucleus pulposus; mTOR, mammalian target of rapamycin; OGT, O-linked N-acetylglucosamine transferase; O-GlcNAc, O-linked β-N-acetylglucosamine.

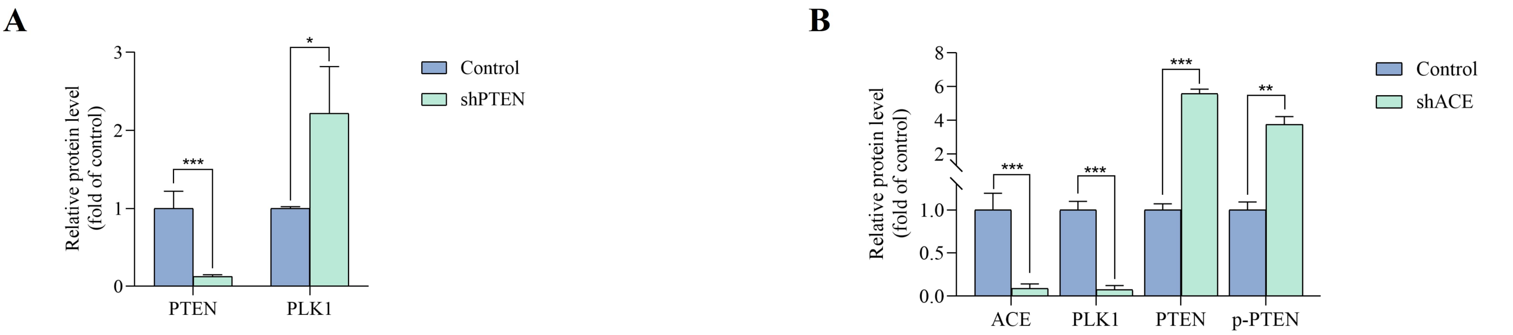

Figure S2 Semi-quantitative analysis of protein after ACE or PTEN knockout. A: NP cells were transduced with the shPTEN for 72 hours. The gray values of protein bands (PLK1 and PTEN) were quantified by ImageJ software (n = 3). B: NP cells were transduced with the shACE for 72 hours. The gray values of protein bands (ACE, PLK1, phosphorylated PTEN, and PTEN) were quantified by ImageJ software. Data was mean ± SD (n = 3). One-way ANOVA and Tukey's multiple comparisons test were used for statistical analysis (n = 3). *$p < 0.05$, **$p < 0.01$ and ***$p < 0.001$. NP, nucleus pulposus; ACE, angiotensin converting enzyme; PTEN, phosphatase and tensin homolog; PLK1, polo-like kinase 1.

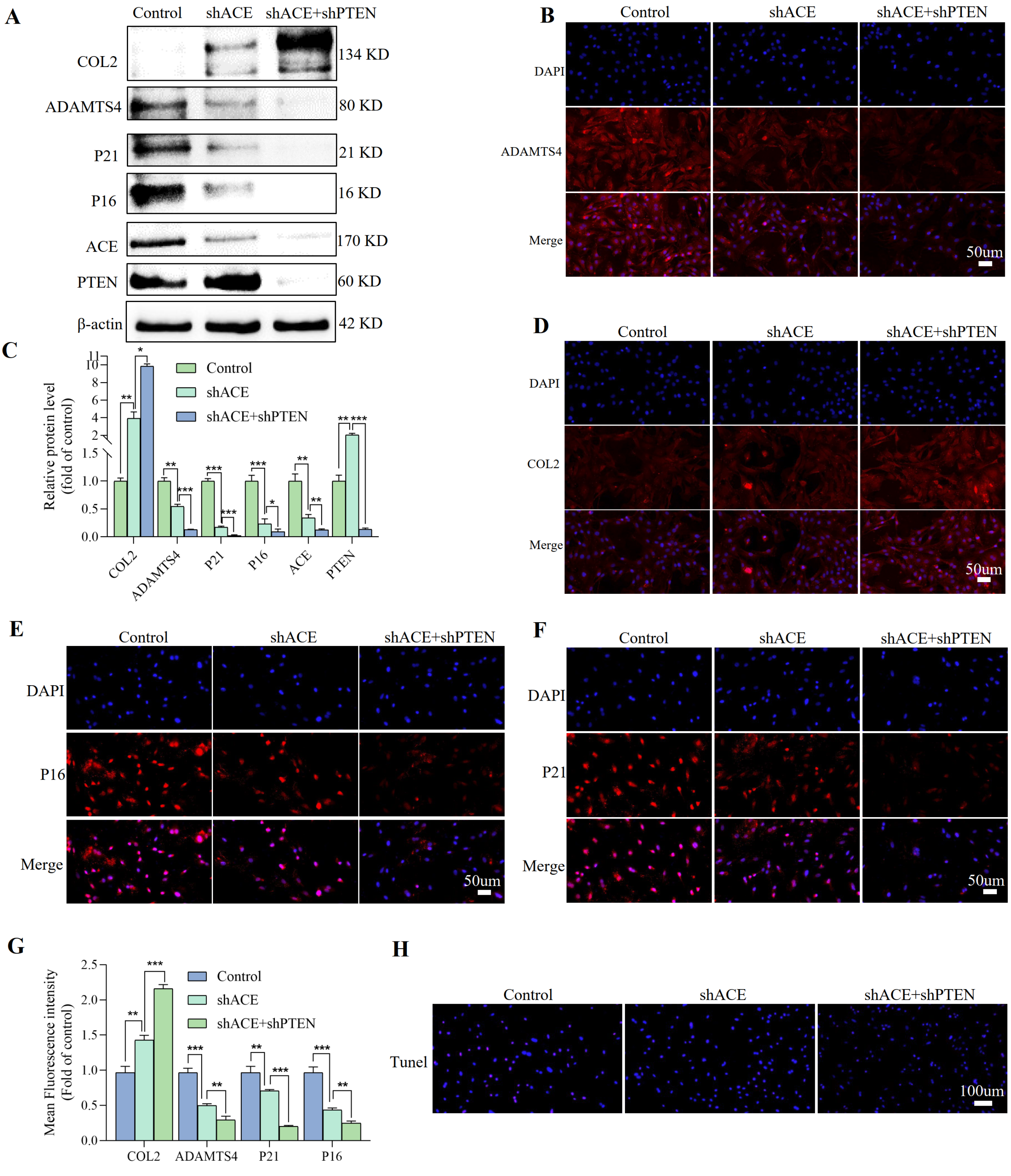

Figure S3 The combined knock-down effect of PTEN and ACE. NP cells underwent transduction with the shACE or combination of shACE and shPTEN for a duration of 72 hours. A and C: Degeneration and senescence-associated proteins COL2, ADAMTS4, p21, and p16 were assessed via western blot analysis, and the relative band density was measured (n = 3). B,D-G: Subsequently, the NP cells were marked with degeneration indicator antibodies (COL2 and ADAMTS4) and senescence antibodies (P21 and P16), exhibiting typical fluorescence pictures. The relative mean optical density was measured (n = 3). Scale bar: 50 μm. H: TUNNEL staining of NP cells was subjected to the indicated shRNA transduction. Scale bar: 100 μm. All experiments were repeated three times. The data are pre-sented as the mean ± SD values. One-way ANOVA and Tukey's multiple comparisons test were used for statistical analysis. *p < 0.05, **p < 0.01, ***p < 0.001. NP, nucleus pulposus; ACE, angiotensin converting enzyme; PTEN, phosphatase and tensin homolog; COL2, Collagen II; ADAMTS4, a disintegrin and metalloproteinase with thrombospondin motif 4.

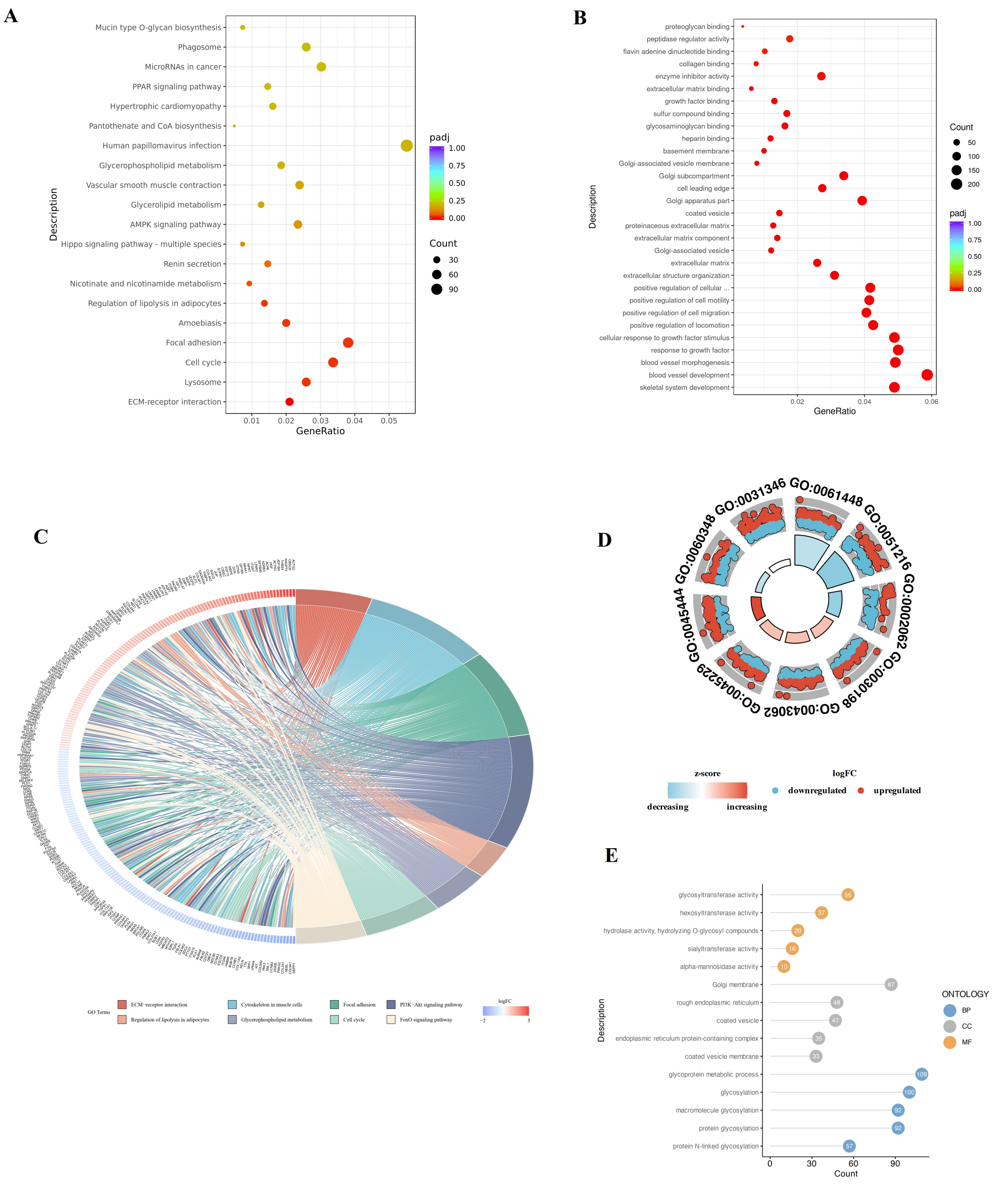

Figure S4 GO and KEGG pathway enrichment analysis. A and B: Dot or Bubble charts of the KEGG and GO enrichment terms. C: Chordal graph of the DEGs to KEGG pathways. The width of the chordal represents the log2FC value. D: GO enrichment circle dia-gram ($p \leq 0.05$). E: Lollipop chart of the GO enrichment terms. KEGG, Kyoto encyclopedia of genes and genomes; GO, Gene ontology.

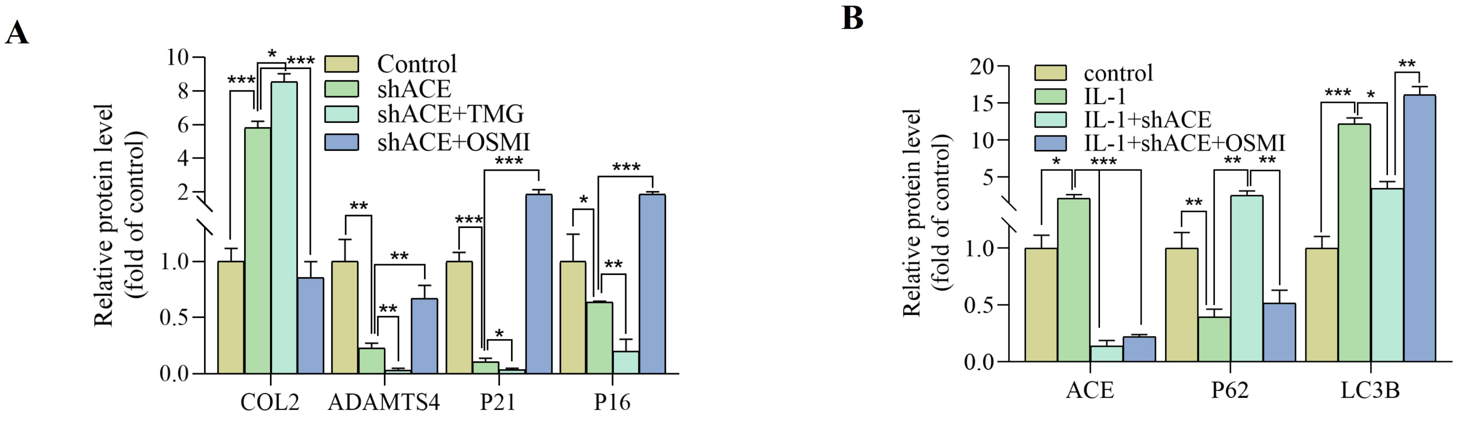

Figure S5 Quantification of the effect of glycosylation regulated by ACE on ER autophagy. A: Forty-eight hours after shACE transfection, NP cells were treated with the TMG (10 μM) or OSMI (25 μM) thirty-six hours. The gray values of pro-tein bands (COL2, ADAMTS4, p21, and p16) were quantified by ImageJ software (n = 3). B: The NP cells treated with IL-1 (10 ng/ml) were transfected with shACE. The NP cells transfected with shACE were treated with OSMI (25 μM) or not. The gray values of pro-tein bands (P62 and LC3B) were quantified by ImageJ software (n = 3). Data was mean ± SD (n = 3). One-way ANOVA and Tukey's multiple comparisons test were used for statistical analysis. *p < 0.05, **p < 0.01 and ****p < 0.001. NP, nucleus pulposus; ACE, angiotensin converting enzyme; ER, endoplasmic reticulum; TMG, Thiamet G; OSMI, O-GlcNAc transferase inhibitor; COL2, Collagen II; ADAMTS4, a disintegrin and metallo-proteinase with thrombospondin motif 4.

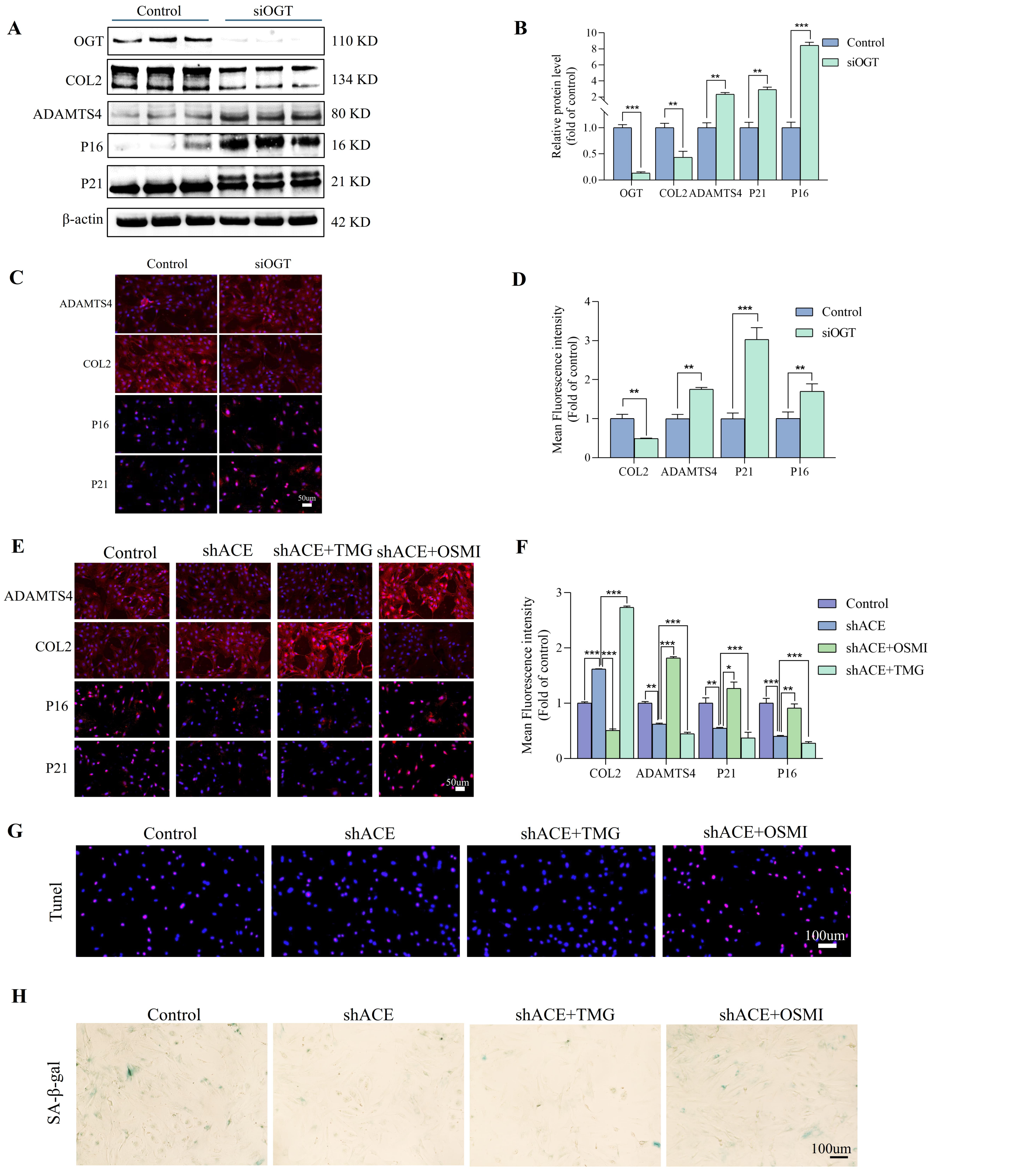

Figure S6 The functional verification of OGT and the effect of glycosylation regulated by ACE on ER autophagy. A-D: NP cells were transduced with OGT siRNA for 72 hours. Degeneration and senescence-associated proteins COL2, ADAMTS4, p21, and p16 were evaluated using western blot analysis and immunofluorescence labeling. Fluorescence intensity and band density were assessed by semiquantitative analysis (n = 3). Scale bar: 50 μm. E and F: Forty-eight hours after shACE transfection, NP cells were treated with the TMG (10 μM) or OSMI (25 μM) thirty-six hours. Degeneration and senescence-associated proteins COL2, ADAMTS4, p21, and p16 were evaluated using immunofluorescence labeling. Fluorescence intensity was assessed by semiquantitative analysis(n = 3). Scale bar: 50 μm. G and H: TUNNEL and β-galactosidase staining of NP cells with above treatments. Scale bar: 100 μm. All experiments were repeated three times. All data are expressed as the mean ± SD. One-way ANOVA and Tukey's multiple comparisons test were used for statistical analysis. *p < 0.05, **p < 0.01, ***p < 0.001. NP, nucleus pulposus; ACE, angiotensin converting enzyme; COL2, Collagen II; TMG, Thiamet G; OSMI, O-GlcNAc transferase inhibitor; ADAMTS4, a disintegrin and metallo-proteinase with thrombospondin motif 4; OGT, O-GlcNAc Transferase.

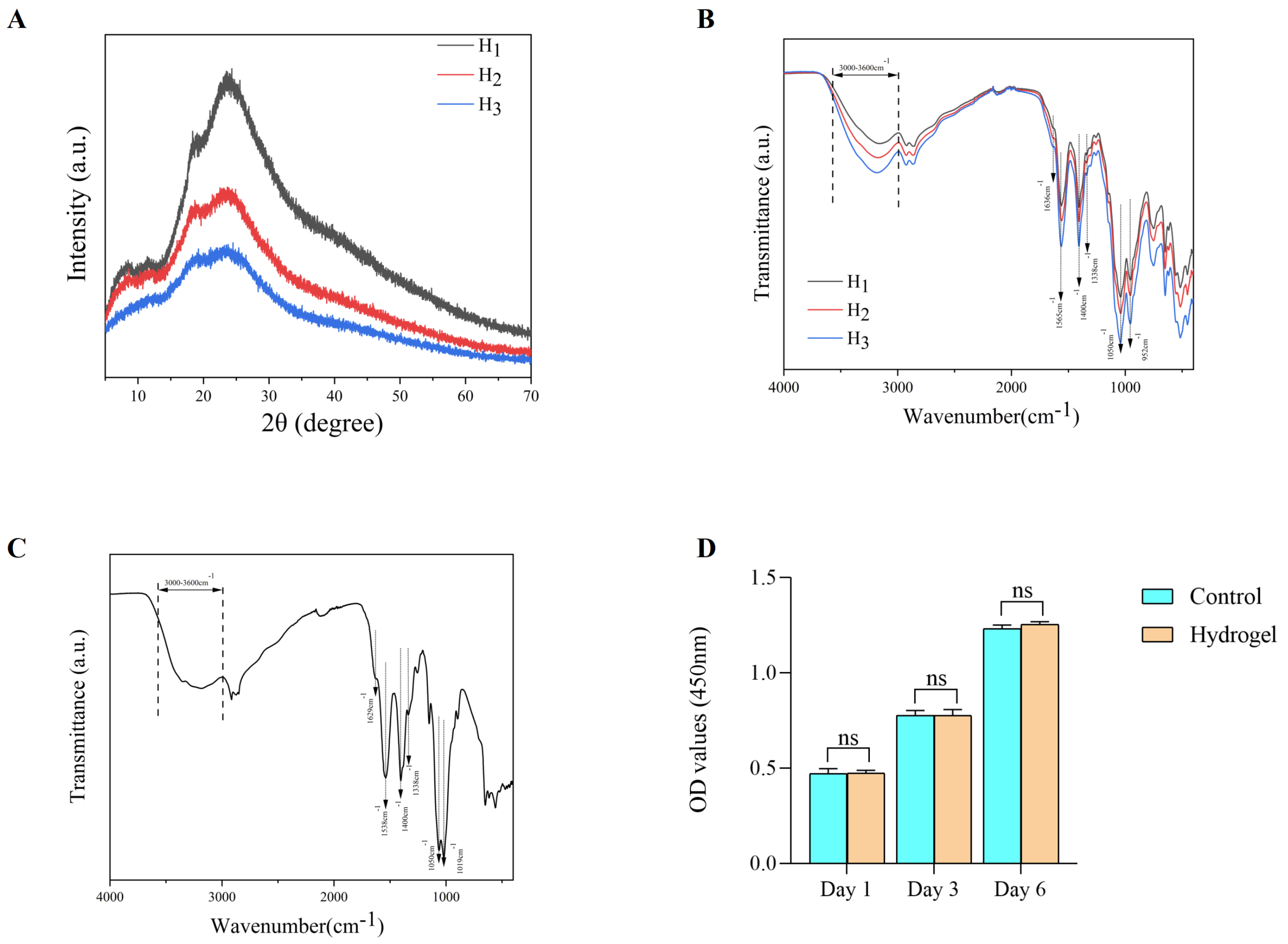

Figure S7 Physical and biological properties of hydrogels. A: XRD patterns of the prepared hydrogels (H1, H2, and H3). In the XRD pattern, all hydrogel samples showed wide peaks of chitosan near 21˚. B: ATR-FTIR spectra of the prepared hydrogels (H1, H2, and H3). In the ATR-FTIR spectrum, the stretching vibration absorption bands of chitosan O-H and -NH2 were found at 3000-3600 cm^{-1}, and the intermolecular and intramolecular hydrogen bonding made the bands wider. 1633 cm^{-1} was the C=O stretching vibration absorption peak, and 1565 cm^{-1} was the N-H deformation vibration absorption peak. 1400 cm^{-1} is the bending vibration absorption peak of C-H, 1338 cm^{-1} is the stretching vibration absorption peak of C-N, and 1050 cm^{-1} is the stretching vibration absorption peak of C-O. And 952 cm^{-1} is the symmetric stretching vibration absorption peak of PO43^{-}. C: ATR-FTIR spectra of chitosan. Compared with CS, it was found that C=O and N-H in composite hydrogel showed obvious redshifts (1629 cm^{-1}→1633 cm^{-1}, 1538 cm^{-1}→1565 cm^{-1}), indicating that hydrogen bonds and complexes were formed between CS molecular chain and β-GP. The molecular force between CS and β-GP forms a three-dimensional network, which is the main reason for the formation of gels in addition to the regulation of pH by β-GP. D: CCK-8 cell viability assay of NP cells co-cultured with hydrogel (H1) for 1 day to 6 days. ATR-FTIR, attenuated total reflection Fourier transform Infrared Spectroscopy; XRD, X-ray Diffraction; CS, Chitosan; β-GP, β-Glycerophosphate; NP, nucleus pulposus.

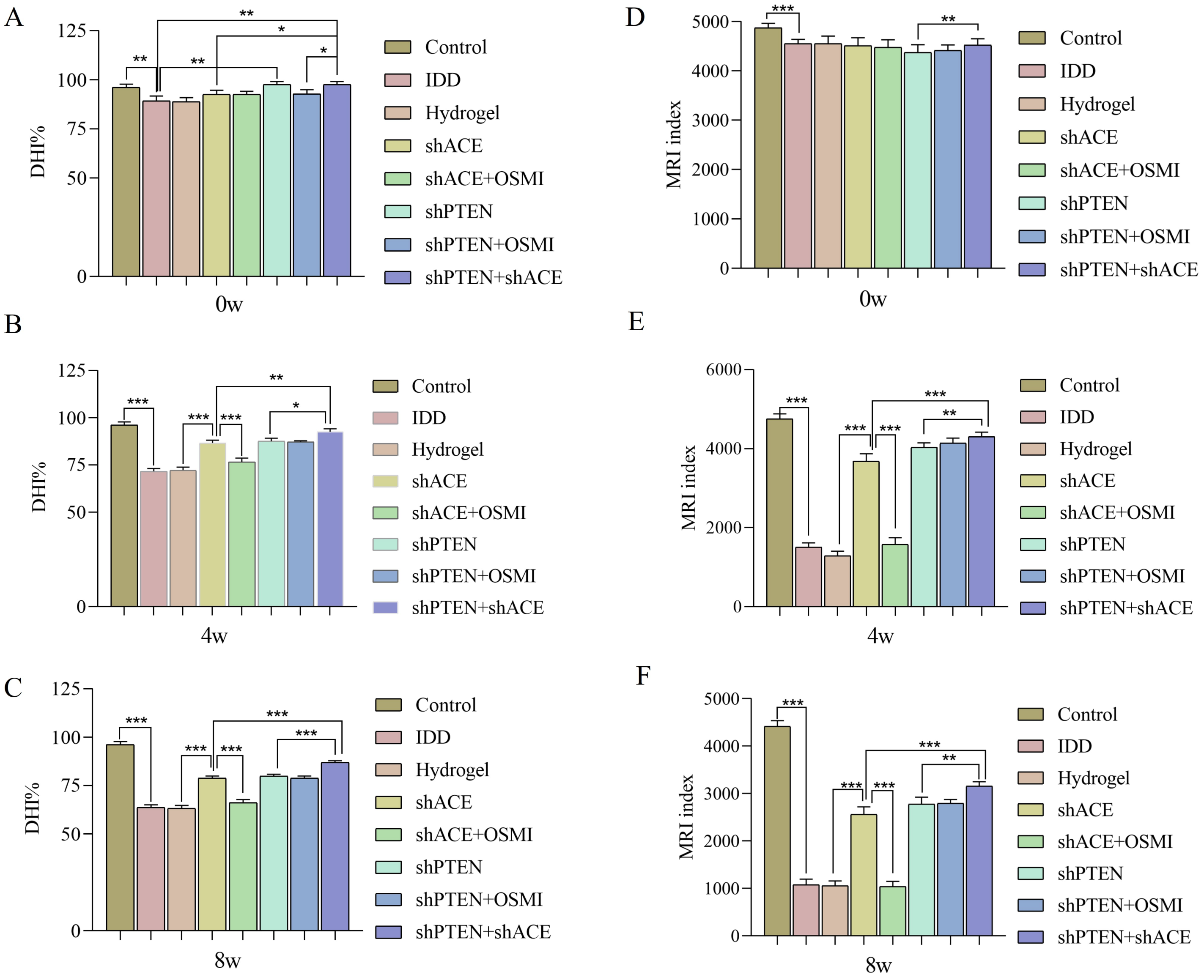

Figure S8 Exponential quantitative calculation of IVD and radiographic evaluations of rat tail. A-C: Diagram represented the disc height changes at 0, 4 and 8 weeks after the intervention. D-F: MRI index changes of each group at 0, 4 and 8 weeks after the intervention. All experiments were repeated ten times. All data are expressed as the mean ± SD. One-way ANOVA and Tukey's multiple comparisons test were used for statistical analysis. *$p < 0.05$, **$p < 0.01$, ***$p < 0.001$. IVD, intervertebral disc; MRI, magnetic resonance imaging; DHI, disc height index; ACE, angiotensin converting enzyme; PTEN, phosphatase and tensin homolog; OSMI, O-GlcNAc transferase inhibitor; IDD, intervertebral disc degeneration.
